# Barriers and facilitators for female practitioners in orthopaedic training and practice: a scoping review

**DOI:** 10.1111/ans.19334

**Published:** 2025-01-03

**Authors:** Clara Freeman, Rebecca Evans, Natalie Drever, Jordy White, Sarah Larkins, Christopher Morrey

**Affiliations:** ^1^ College of Medicine and Dentistry James Cook University Cairns Queensland Australia; ^2^ College of Medicine and Dentistry James Cook University Townsville Queensland Australia; ^3^ Department of Obstetrics and Gynaecology Cairns Hospital Cairns Queensland Australia; ^4^ Department of Orthopaedics Cairns Hospital Cairns Queensland Australia

**Keywords:** orthopaedics, diversity, equity, female, inclusion, orthopaedic surgeons, women, workforce diversity

## Abstract

**Background:**

Despite advances in medical education and professional opportunities, orthopaedic surgery remains the least gender‐diverse medical specialty, with women significantly underrepresented globally. This scoping review aims to synthesize existing literature to provide a comprehensive overview of the barriers and facilitators encountered by females in orthopaedic surgery training and practice.

**Methods:**

A comprehensive search of Medline (OVID), Scopus, Embase, Emcare, and CINAHL was performed from inception to 14 July 2024. Additional sources were identified via citation searching and Google Scholar. Any primary studies employing qualitative, quantitative, or mixed methods approaches to explore barriers and facilitators experienced by female orthopaedic trainees and consultants in high‐income countries. Quality analysis of included articles was conducted using the Mixed Methods Appraisal Tool.

**Results:**

Seventy‐nine studies met the inclusion criteria, involving over 100 000 participants between 1993 and 2024. Most studies were cross‐sectional surveys. Sixty‐eight barriers and 38 facilitators were identified. Analysis using the Socio‐Ecological Model revealed the complex interplay of factors at the individual, interpersonal, organizational, community, and policy levels. The largest proportion of barriers and facilitators resided at the organizational level.

**Conclusion:**

This scoping review provides a comprehensive mapping of current evidence on barriers and facilitators for female practitioners in orthopaedic surgery training and practice. The findings suggest the need for multifaceted interventions to promote gender equity. Future research should evaluate the effectiveness of specific interventions and develop strategies to support women in orthopaedics, ultimately contributing to a more inclusive and diverse workforce.

## Graphical abstract

This scoping review synthesises existing literature to identify barriers and facilitators for female practitioners in orthopaedic surgery, globally. The analysis, based of 79 included studies involving over 100,000 participants, highlights a significant proportion of barriers and facilitators at the organisational level of the Socio‐Ecological Model. Findings underscore the need for multifaceted strategies to promote gender equity in the field.

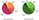

## Introduction

Around the world diversity and inclusion is progressing exponentially. This is seen in many professional domains, including the recent Paris 2024 Olympics being the first with an equal allocation of male and female athletes.^1^ In healthcare teams, achieving critical mass diversity improves patient care quality as diverse medical teams are associated with more accurate diagnoses, higher patient satisfaction and better health outcomes.^2^ Interpersonally, diversity enhances professional skills such as communication, teamwork, and efficiency.^2^ Furthermore, diversity improves innovation, attracts top talent and improves financial performance by boosting revenues and creating superior risk assessments.^2^ Diversity spans many important realms including. but not limited to, culture, gender, sexual orientation, disability, age and religion. This review will focus only on gender diversity.

In many countries, women represent over half the medical students graduating university.^3^ However, as described by the International Orthopaedic Diversity Alliance female practitioners aside from six countries (Estonia, Sweden, Brunei, Canada, Colombia and Malaysia), women constitute less than 10% of the orthopaedic surgeons worldwide. The United States of America (USA) had 6.1%, New Zealand 5.0%, Japan 4.9%, United Kingdom (UK) 4.8% and Australia sat at 16th with 4.3% of the orthopaedic surgeons being female.^3^

Gender parity is achieved when critical mass has been reached. Currently, no country has reached this in orthopaedics.^3^, ^4^ Critical mass in diversity was first noted in 1977, in which Kanter introduced the idea that once women and minorities reach a certain threshold in an organization, their presence can influence the overall dynamics and culture of the group, making it more inclusive and equitable.^4^ Since then, the critical mass point is widely accepted to be 30%, and quantities below this is considered to lead to tokenism.^5^ Notably, studies estimate from 2024 it will take over 212 years in the USA,^6^ 46 years in the UK,^7^ 156 years in Japan^8^ and 185 years in Australia^9^ to reach gender parity in orthopaedic surgery if trends remain the same.

Extensive literature exists on gender diversity in healthcare and within the specialty of orthopaedic surgery. Numerous narrative reviews and editorials broadly discuss the barriers women face in orthopaedics.^3^, ^10^, ^11^, ^12^, ^13^, ^14^ Systematic reviews have also addressed specific barriers such as mentoring,^15^ family planning and pregnancy,^16^ medical school experiences,^17^ and bullying and harassment.^18^ A scoping review is needed to synthesize this fragmented information into a comprehensive, approachable overview.

The objective of this scoping review is to systematically identify and map the existing literature regarding barriers and facilitators to females training and practicing in orthopaedic surgery. To our knowledge this is the first scoping review to do this. The findings of this review aims to equip prospective trainees, clinicians, and stakeholders with a thorough understanding of the challenges and supports, ultimately guiding effective strategies, interventions and policy change to promote gender diversity and an inclusive environment in orthopaedic surgery.

## Methods

### Protocol and registration

This scoping review was conducted using the enhanced Arksey and O'Malley framework^19^ and analysed using the Socio‐Ecological Model (SEM).^20^ The review utilizes the Preferred Reporting Items for Systematic Reviews and Meta‐Analyses with extension for Scoping Reviews (PRISMA‐ScR).^21^ The review protocol has been registered with Open Science Framework Registry and can be accessed at DOI: 10.17605/osf.io/uebgm/.

### Eligibility criteria

Inclusion and exclusion of studies was based on the ‘Population, Intervention, Comparator, Observation’ (PICO)^22^ table outlined in Table S1. Primary studies employing qualitative, quantitative, or mixed methods approaches were included to explore barriers and facilitators experienced by female orthopaedic trainees and consultants in high‐income countries. High‐income countries, classified by the World Bank, were exclusively included to ensure comparability in healthcare systems, professional opportunities, and sociocultural contexts. Reviews, editorials, opinion pieces, studies focused on medical student perspectives, orthopaedic subspecialties, and articles concentrating solely on statistical demographics without exploring causation or implications of gender disparity were excluded. No language limitations were applied. Google Translate was used to translate non‐English articles.

### Information sources

A comprehensive search was conducted in Medline (OVID), Scopus, Embase, Emcare, and CINAHL from inception to the most recent search on 14 July 2024. Additional sources included citation searching and Google Scholar.

### Search

The full electronic search strategy is presented in Table S2. A combination of keywords and Medical Subject Headings (MeSH) terms related to ‘female’ AND ‘doctor’ AND ‘orthopaedic surgery’ was used. The search strategy was tailored to each database.

### Selection of sources of evidence

The selection process involved two stages; initial screening of titles and abstracts, followed by full‐text review. Two reviewers (*CF* and ND) independently screened the sources of evidence for eligibility. Discrepancies were resolved by a third reviewer (JW). Covidence software^23^ was used to maintain integrity in the review process and to automatically identify and remove duplicates efficiently. This was supplemented by manual duplicate removal where required.

### Data charting process

Data was charted using a standardized template developed by *CF* and ND. The form was calibrated through pilot testing, where *CF* charted, and ND reviewed it to ensure accuracy and consistency. Discrepancies were resolved through discussion. The subsequent data charting was completed by CF.

### Data items

Data was extracted on the following variables: author, title, year, country, study design, setting, aim, method, population, sample size, response rate, female sample proportion, results, barriers, facilitators, limitations and discussion.

### Critical appraisal of individual sources of evidence

The Mixed Methods Appraisal Tool (MMAT, Table S3)^24^ was utilized for quality assessment of included articles. *CF* and ND independently assessed the articles to ensure meticulous accuracy.

The MMAT rates the methodological quality of studies using two screening questions followed by five questions, relevant to on the study design, requiring a ‘Yes’, ‘No’ or ‘Can't Tell’ response. Each of the five study design categories: qualitative, quantitative randomized controlled trials, quantitative non‐randomized controlled trials, quantitative descriptive and mixed methods, has a unique set of criteria questions.^24^

MMAT discourages exclusion of low quality studies and encourages a sensitivity analysis where results of studies are contrasted based on their quality. Studies which source a ‘Yes’ across all five criteria are considered higher quality, than those which receive ‘No’ or ‘Can't Tell’ responses on criteria.^24^

### Synthesis of results

Data was synthesized using descriptive and thematic analysis. The findings were then mapped to the Socio‐Ecological Model (SEM). The SEM has been applied to understand a number of health‐related phenomena (insert references) and structures consideration^25^, ^26^, ^27^ across a number of levels, including individual, interpersonal, organizational, community and policy factors.^20^ This approach helps to identify how these different levels interact and impact the experience of female orthopaedic surgeons, offering a holistic view of the challenges and supports within the professional environment. The SEM's comprehensive framework provides insights into the multifaceted interplay of factors affecting gender equity in orthopaedic surgery.

## Results

### Selection of sources of evidence

**Fig. 1:**
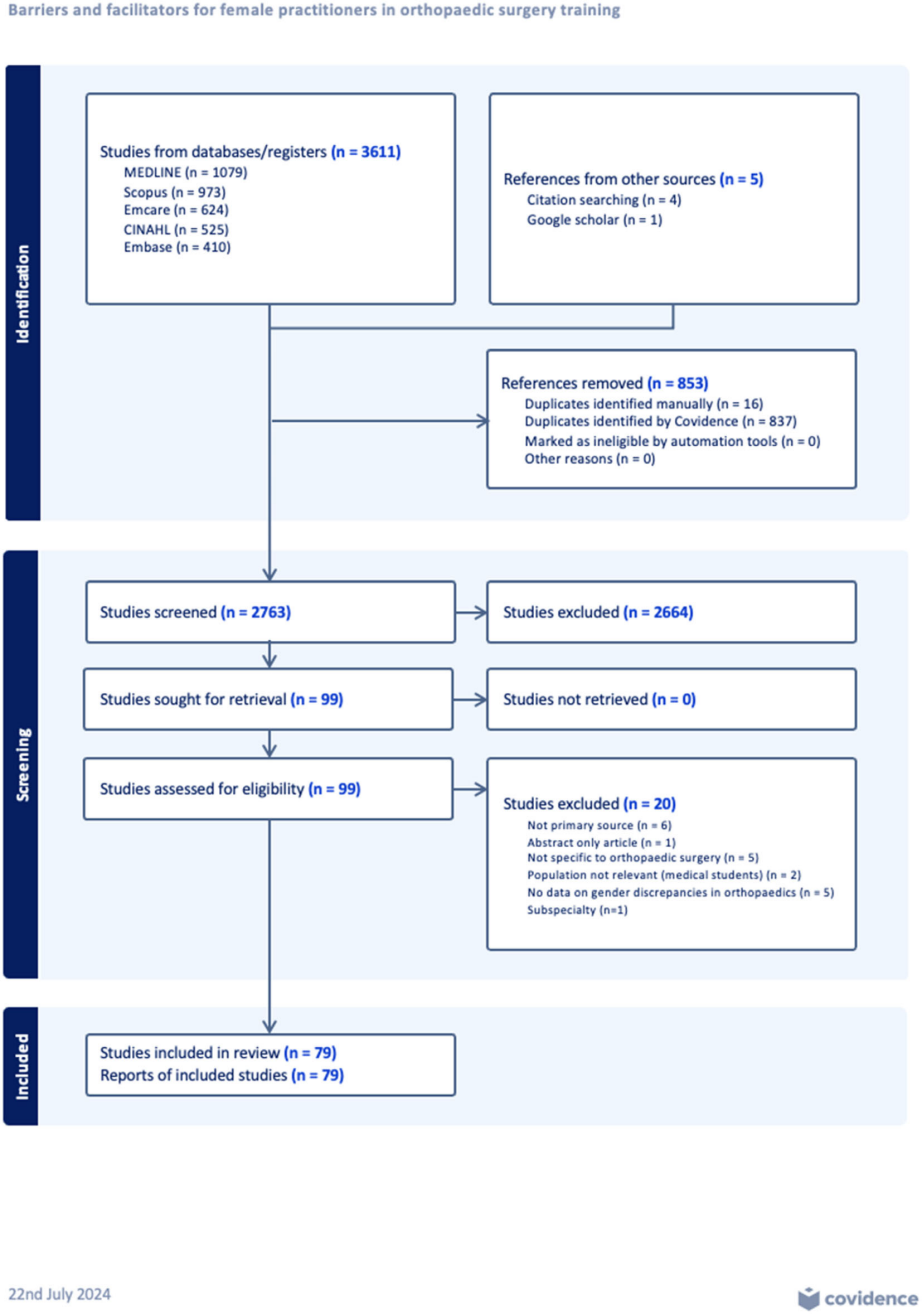
Preferred reporting items for systematic reviews and meta‐analyses with extension for scoping reviews (PRISMA‐ScR).^21^

A total of 3611 sources of evidence were identified from the database searches and 5 from additional sources. After removing duplicates and title/abstract screening, 99 sources were selected for full text review out of which 79 met eligibility criteria. Fig. 1 shows the Covidence software adapted PRISMA‐ScR flow chart.^21^ Search strategies and excluded studies are presented in Tables S2 and S4, respectively.

### Characteristics of sources of evidence

The 79 included studies were published between 1993 and 2024 and involved over 100 000 participants. Majority of studies were cross‐sectional surveys (*n* = 37), followed by retrospective studies (*n* = 19), cross‐sectional analyses (*n* = 14), mixed methods (*n* = 7), and qualitative studies (*n* = 2). Characteristics of included studies are summarized in Table 1 and detailed further in Table S5. Half of the publications were within the last 4 years, 2021–2024 (40/79, 50.63%), closely followed by the prior 5 years from 2016 to 2020 (32/79, 40.51%). Therefore over 90% of the included studies on the topic have been published within the preceding 10 years. The majority of articles originated from the USA (*n* = 63), followed by Canada (*n* = 5), the UK (*n* = 3), the Gulf Cooperation Council Countries (*n* = 2). Additionally, there was one article identified from each of Argentina, Australia, Belgium, Israel, New Zealand and South Africa.

**Table 1:** Summary of characteristics of included studies

Characteristics	Values	Number (%) of studies
Year of publication	1990–1995	1 (1.27)
	1996–2000	1 (1.27)
	2001–2005	0 (0.00)
	2006–2010	1 (1.27)
	2011–2015	4 (5.06)
	2016–2020	32 (40.51)
	2021–2024	40 (50.63)
Country of conduct	United States of America	63 (79.75)
	Canada	5 (6.33)
	United Kingdom	3 (3.80)
	Gulf Cooperation Council Countries	2 (2.53)
	Argentina	1 (1.27)
	Australia	1 (1.27)
	Belgium	1 (1.27)
	Israel	1 (1.27)
	New Zealand	1 (1.27)
	South Africa	1 (1.27)
Design	Qualitative	2 (2.53)
	Retrospective studies	19 (24.05)
	Cross sectional analysis	14 (17.72)
	Cross sectional survey	37 (46.84)
	Mixed methods	7 (8.86)

### Results of individual sources of evidence

The study characteristics, participant details and list of barriers and facilitators identified is detailed in Table S5. Those barriers and facilitators were mapped to the SEM in Table S6.

### Synthesis of results

Of the included studies, 70 studies (88.61%) identified barriers, and 50 studies (63.29%) identified facilitators. There were 68 different barriers identified (68/107, 63.55%), 38 facilitators (38/107, 35.51%) and 1 factor which was not classed as either a barrier or facilitator (1/107, 0.93%).

**Fig. 2:**
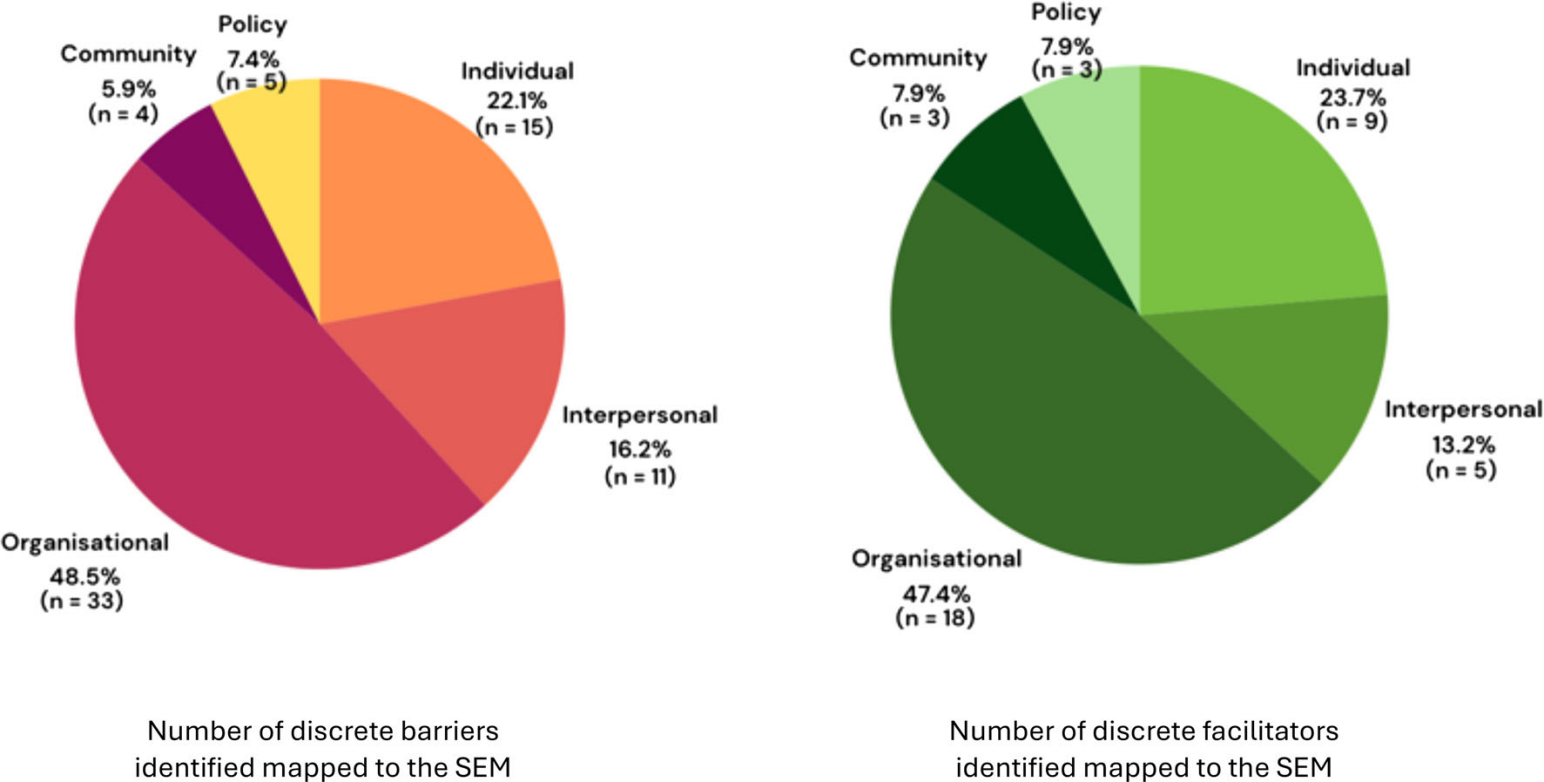
Visual representation of the number of discrete barriers and facilitators identified and mapped to the socio‐ecological model.

Both barriers and facilitators were represented across the five SEM levels with the majority of barriers and facilitators being categorized at the organizational level. The dispersion of identified barriers and facilitators are visually represented in Fig. 2.

Further, barriers were more commonly reported than facilitators: barriers were identified on 269 (269/399, 67.42%) occasions amongst the articles and facilitators identified on128 occasions (128/399, 32.08%). The non‐classified factor was identified on 2 occasions (2/399, 0.50%). When broken down using the SEM, articles were identified to discuss barriers at the individual level 47 times (47/269, 17.47%), interpersonal level 52 times (52/269, 19.33%), organizational level 131 times (131/269, 48.70%), community level 23 times (23/269, 8.55%) and policy level 16 times (16/269, 5.95%). Whilst articles identified facilitators at the individual level 26 times (26/128, 20.31%), the interpersonal level 17 times (17/128, 13.28%), organizational level 64 times (64/128, 50.00%), community level 5 times (5/128, 3.91%) and policy level 16 times (16/128 12.50%). Details on the barriers and facilitators on each level of the SEM are outlined below.

## Barriers

### Individual level

Individual level barriers identified for female practitioners in orthopaedic surgery were subcategorised into psychological, personal life and physical classes. Psychological barriers women faced included imposter syndrome,^28^, ^29^, ^30^ lower self‐assessment,^31^, ^32^ performance anxiety,^32^, ^33^ psychological distress,^34^ burnout,^34^, ^35^, ^36^, ^37^ and pride fatigue in challenging the status quo.^29^ Within their personal life female orthopaedic surgeons reported being in married/committed relationships less than their male colleagues.^30^, ^37^, ^38^, ^39^, ^40^, ^41^ Female orthopaedic surgeons are also less commonly parents compared to their male counterparts.^37^, ^38^, ^39^, ^40^, ^41^ They also reported lower levels of marital harmony,^34^ can have unsupportive partners,^32^, ^34^ and require longer parental leave.^42^, ^43^, ^44^ Most female orthopaedic surgeons deferred pregnancy,^29^, ^43^, ^45^, ^46^ leading to infertility and complications.^29^ Further, physical difficulty associated with pregnancy such as morning sickness,^28^, ^44^ reportedly impacted scholarly activities.^29^, ^43^, ^45^, ^47^ Additionally, the physical demands of orthopaedics can deter women from entering the profession.^37^, ^38^, ^41^, ^48^, ^49^, ^50^, ^51^

### Interpersonal level

Female orthopaedic surgeons struggled with the lack of female role models and mentors available to them.^28^, ^30^, ^32^, ^41^, ^48^, ^49^, ^51^, ^52^ Women reported facing negative workplace culture including microaggressions daily,^53^, ^54^, ^55^ bullying,^28^, ^56^ sexual harassment,^28^, ^30^, ^34^, ^52^, ^54^, ^56^, ^57^, ^58^ gender based harassment,^57^ and social exclusion.^28^, ^29^, ^30^, ^55^ Additionally, negative attitudes and perceptions by surgical colleagues,^28^, ^29^, ^30^, ^37^, ^43^, ^45^, ^46^, ^47^, ^48^, ^51^, ^55^, ^59^, ^60^ differential treatment from other hospital staff such as nurses, allied health and wards people^28^, ^29^, ^37^, ^54^, ^55^ emerged as key barriers. Instances included female surgeon's directives being questioned or not followed with the same immediacy or respect as their male counterparts and environments in which female surgeons are perceived as needing to earn respect through deference, contrasting with the automatic authority often granted to male surgeons.^28^, ^29^, ^37^, ^54^, ^55^ Role misidentification,^29^, ^55^ women being interrupted more when speaking,^29^, ^54^ and the perceived burden pregnancy places on co‐workers^29^, ^43^, ^45^, ^47^ also impacted female orthopaedic surgeons.

### Organizational level

Majority of the barriers female orthopaedic surgeons face reside at the organizational level. Female surgeons face an ongoing male‐dominated culture^28^, ^35^, ^38^, ^51^, ^52^ in orthopaedics which can cultivate gender discrimination,^34^, ^36^, ^37^, ^51^, ^55^, ^56^, ^61^ gender stereotypes such as female surgeons being labelled bossy, demanding and difficult rather than assertive, decisive and a strong leader,^29^, ^32^, ^36^, ^55^, ^62^ and workplace violence, assaults and threats.^63^ Career advancement barriers identified included a lack of exposure to orthopaedics,^51^, ^64^ lack of gender diverse faculty,^41^, ^52^, ^60^, ^65^, ^66^, ^67^, ^68^ higher attrition rates for women,^30^, ^36^, ^46^, ^69^, ^70^, ^71^, ^72^ gender‐based inequality to promotion,^28^, ^30^, ^36^, ^37^, ^39^, ^52^, ^73^ gender discrepancy in speaker roles at conferences,^30^, ^55^, ^68^, ^74^, ^75^ gender imbalances in leadership roles and committees,^28^, ^30^, ^40^, ^52^, ^68^, ^74^, ^75^, ^76^, ^77^, ^78^ underrepresentation and implicit bias in leadership awards,^79^ disproportionate research funding allocation,^80^ and receiving less financial reimbursement for the work completed.^29^, ^40^, ^80^, ^81^, ^82^, ^83^, ^84^, ^85^ Within the working environment, inappropriate interview questions,^29^, ^49^, ^54^, ^60^, ^86^, ^87^ affinity bias in letters of recommendation to training programs,^88^, ^89^, ^90^ lower training application scores of women,^90^, ^91^, ^92^, ^93^ gender‐based disparity in operative autonomy in training,^37^, ^42^ lower service volume and diversity of practice,^37^, ^81^, ^82^ disproportionate constraints,^28^, ^35^, ^36^, ^37^, ^54^, ^55^ devaluation,^28^, ^29^, ^34^, ^35^, ^36^, ^37^, ^55^ tokenism,^29^, ^55^ gendered task assignment,^28^, ^54^, ^55^ gendered terms such as “chairman,”^94^ and limited support networks^28^, ^30^, ^46^ emerged as key barriers. The financial burden of maternity leave^45^, ^95^ and increased average time taken off for teaching, research and maternity leave^40^, ^42^, ^45^, ^95^ were identified as barriers. Plus, women encounter deficient locker rooms, well‐fitting lead shielding, milk production and storage facilities and childcare options.^43^, ^44^, ^46^, ^47^, ^55^, ^67^, ^96^

### Community level

Barriers identified at the community level included patient inflicted bias,^28^, ^29^, ^52^, ^54^, ^55^ limited visibility for female orthopaedic surgeons,^68^, ^75^, ^79^ and societal norms and attitudes that women hold a greater role in household work^30^, ^32^, ^39^, ^40^, ^52^ and childrearing responsibilities^28^, ^29^, ^30^, ^32^, ^39^, ^40^, ^44^, ^46^, ^51^, ^52^ than men.

### Policy level

A lack of adequate policy and policy awareness around breastfeeding,^96^ maternity leave,^28^, ^29^, ^43^, ^44^, ^45^, ^46^, ^47^, ^95^ discrimination, bullying and sexual harassment,^56^, ^58^ coupled with ineffective reporting streams^50^, ^56^, ^58^, ^86^ were identified as barriers. Additionally, ill‐designed surgical instruments causing ergonomic challenges for smaller hands was a barrier.^50^

## Facilitators

### Individual level

Throughout the included studies individual psychological factors of personal interest,^37^, ^38^, ^49^, ^61^ career satisfaction,^29^, ^32^, ^35^, ^36^, ^37^, ^38^, ^40^, ^41^, ^61^, ^62^ desire to teach^30^, ^32^ and orthopaedics being an intellectual challenge^32^, ^37^, ^41^ were common enablers. Personal life facilitators included having a personal background as a sportsperson,^49^ a family member who practices surgery,^49^ a supportive partner^44^ and pushing back against inequality.^29^, ^44^ Lastly many women who pursued orthopaedics reported to enjoy manual tasks.^38^, ^41^

### Interpersonal level

Support systems are key interpersonal facilitators for female orthopaedic surgeons, most significantly mentorship,^29^, ^30^, ^32^, ^41^, ^48^, ^51^, ^73^, ^97^, ^98^, ^99^ followed by networking,^97^ and supportive colleagues.^44^ Inclusion in sporting and social events^30^ and trainee comradery^30^, ^60^, ^65^, ^100^ also appeared to support women in orthopaedics.

### Organizational level

As with barriers, majority of the facilitators for women in orthopaedic surgery lie at the organizational level. Assistance to career advancement includes early exposure to orthopaedics,^41^, ^48^, ^51^, ^61^, ^64^, ^98^, ^99^ pipeline programs,^30^, ^98^, ^99^ positive medical school experiences in orthopaedics,^38^, ^48^, ^60^, ^61^, ^64^, ^98^, ^100^ clinical opportunities in training,^65^ women in leadership positions and on committees,^30^, ^68^, ^74^, ^77^, ^78^, ^101^, ^102^ having an academic practice^30^, ^49^, ^78^ and on average women received higher interview scores.^91^, ^93^ Appreciably, 12 studies highlighted the importance of enhanced gender diversity fostering further gender equality.^60^, ^66^, ^71^, ^75^, ^76^, ^78^, ^96^, ^100^, ^101^, ^102^, ^103^, ^104^ Positive staff interactions,^60^, ^99^, ^100^ staff happiness,^60^, ^65^, ^100^ gender neutral language such as chairperson,^94^ onsite childcare,^30^, ^43^, ^45^, ^46^, ^47^, ^95^ facilities to breastfeed, pump and store milk^43^, ^46^, ^47^ all enabled women within the work environment of orthopaedics. Organizational strategies such as using quota systems,^29^ standardized letters of recommendation,^90^ women on training selection panels,^91^ dedicated women's sports medicine programs^78^, ^101^ and blinded award processes^79^ enabled women's selection onto training programs, during programs and in practice as orthopaedic surgeons.

### Community level

At a community level social media, specifically individual surgeons professional and organizations Instagram and Twitter accounts, increases female orthopaedic surgeon's visibility.^97^, ^105^, ^106^ Additionally, being close to social supports^60^ and having patients with positive perceptions^59^ of female surgeons were identified as facilitators.

### Policy level

Facilitators included training programs and hospitals having stated diversity and inclusion efforts or policies,^29^, ^68^, ^74^, ^91^ formalized and standardized maternity or parental leave policies^43^, ^44^, ^45^, ^46^, ^47^, ^78^, ^95^ and availability of flexible scheduling.^30^, ^43^, ^44^, ^47^, ^95^

Lastly, two articles found women were more frequently employed by hospitals rather than private practice, which was not classed as a barrier or facilitator.^40^, ^41^

### Critical appraisal within sources of evidence

The appraisal, utilizing MMAT, revealed varying levels of methodological rigour. 59.47% (47/79) of the included studies met all 5 criteria relevant to their study design and were hence regarded as higher quality. 100% (2/2) of qualitative, 65.63% (21/32) quantitative non‐randomized control trial, 47.37% (18/38) quantitative descriptive and 85.71% (6/7) mixed methods studies were assessed as being of higher quality. No included studies were of the quantitative randomized control trial design. Common limitations encountered included reports on other populations such as orthopaedic program directors and applicants to training programs, a lack of control for confounding variables, failure to state response rates, and the potential presence of nonresponse bias. Further results of the critical appraisal are summarized in Table S7 and detailed in Table S8.

## Discussion

To our knowledge this review is the first of its kind to map the barriers and facilitators experienced by female orthopaedic surgeons in high‐income countries using the SEM to provide a comprehensive systems level view. Most of the literature on this topic utilizes a cross‐sectional survey or analysis design and the literature is dominated by studies originating from North America. There has been exponential growth in the quantity of studies exploring gender diversity in orthopaedics in the past decade, aligning with the contemporary societal push for gender parity in workforces worldwide.

The barriers and facilitators identified in this review imitate those found in prior systematic reviews by Rama *et al*.^10^ and Pechlivanidou *et al*.^14^ The SEM looks at individual, interpersonal, organizational, community and policy levels to identify how the different levels interact and impact society. Importantly, the organizational level held the largest number of identified discrete barriers and occurrences of barriers in articles out of the five SEM levels. As such, much can be actively done by national organizations like the Australian Orthopaedic Association to enhance gender diversity in orthopaedic surgery.

Solutions which minimize barriers and upscale facilitators at an organizational level will likely have knock‐on effects to the interpersonal and individual levels. This is evidenced by Hiemstra *et al*. who demonstrated a positive correlation between burnout, an individual level barrier, to the gender bias factors of male privilege (*r* = 0.215, *P* < 0.01), devaluation (*r* = 0.166, *P* < 0.05) and disproportionate constraints (*r* = 0.152, *P* < 0.05), all organizational barriers.^35^ Additionally, male culture influences other individual level psychological barriers such as imposter syndrome,^28^, ^29^, ^30^ leading to lower self‐assessment.^31^ For example, Brady *et al*. found female orthopaedic surgical participants gave themselves lower scores for all but one training milestone than that which faculty gave. This was despite no significant difference found in medical knowledge or patient care between male and female participants. Comparatively, male participants ranked themselves at or higher than faculty scores.^31^ Therefore, it is essential for organizations to proactively implement strategies which reduce gender biases and foster supportive culture to mitigate the negative impacts on individual well‐being and professional self‐assessment.

Similarly, lower parental rates^37^, ^38^, ^39^, ^40^, ^41^ for women can be addressed by organizational level factors. To avoid female orthopaedic surgeons deferring pregnancy,^29^, ^43^, ^45^, ^46^ adequate maternity, parental and breast feeding policy is required^28^, ^29^, ^43^, ^44^, ^45^, ^46^, ^47^, ^95^, ^96^ and can be influenced by employing organizations. Nguyen *et al*., reported the average leave offered was 4.2–4.6 weeks in the United states but on average 7.4–8.2 weeks were taken, with the average cost of maternity leave for female orthopaedic surgeon being $40 932–$61 258.^95^ In Australia, parental leave can be challenging to navigate due to discrepancies between the organizational requirements of the Australian Orthopaedic Association (AOA), which operates as the training agent for the Royal Australian College of Surgeons, and the employment policies of the state health system.^107^ For example, the AOA may offer a training position interstate, which can disrupt the 40 weeks of uninterrupted employment required for medical officers to qualify for 14 weeks of paid parental leave in their home state.^108^ Additionally, orthopaedic training occurs in 6‐month blocks, which can complicate matters, as pregnancy is unpredictable, and a 6‐month block exceeds the 14 weeks of paid leave, potentially leaving new parents facing unpaid time off.^109^ Consequently, 70% of women, in one study, believe standardized, adequate, prominent parental policies would increase the number of women choosing to pursue orthopaedics.^46^

Interpersonally, negative attitudes and perceptions by surgical colleagues was a key feature in 13 articles.^28^, ^29^, ^30^, ^37^, ^43^, ^45^, ^46^, ^47^, ^48^, ^51^, ^55^, ^59^, ^60^ Tan *et al*. found the lack of acceptance was perceived to be worse in orthopaedics than in general surgery in New Zealand.^51^ This can deter women from the field and can lead to women leaving orthopaedic training without obtaining all technical skills.^30^ Downie *et al*. found in the United Kingdom, upon graduating, male trainees were lead surgeon on 3% more cases then female trainees, resulting in men performing 54 more lead operative cases during their training, potentially impacting their experience level, confidence and career progression in orthoapedics.^42^

Organisationally, limited visibility of female orthopaedic surgeons leads to limited role models and mentors for prospective female surgeons.^28^, ^30^, ^32^, ^41^, ^48^, ^49^, ^51^, ^52^ Vivekanantha *et al*. reported 58.5% of speaker sessions where male only panels at conferences^68^ and Ramos *et al*. found 52.1% of societies in the USA had never had a female president.^77^ Social media engagement with #ilooklikeasurgeon plays a part in enabling visibility.^97^, ^105^, ^106^ However, most notably 12 articles pointed to enhanced gender diversity fostering more diversity,^60^, ^66^, ^71^, ^75^, ^76^, ^78^, ^96^, ^100^, ^101^, ^102^, ^103^, ^104^ this is the same concept of critical mass.^4^ More female orthopaedic surgeons on training programs, as board members and speaking at conferences, increases the number of women in training. Increased visibility is currently being undertaken by various organizations outlined in the systematic review by Rama *et al*.^10^

This review has highlighted the crucial role of the organizational level in shaping experiences of female practitioners in orthopaedic surgery. This is important as outlined by a study published in the Harvard Business Review in 2019, gender diversity leads to increased productivity, better problem solving within teams, increased innovation, and improved financial performance of organizations.^110^ Specific to healthcare Gomez *et al*. found diverse treating teams are associated with more accurate diagnoses, higher patient satisfaction and better health outcomes.^2^

In the Australian setting, since the Australian Orthopaedic Association's implementation of their diversity strategy, affinity bias was decreased at the interview stage of the training application process.^91^ Plus, Australia utilizes the current gold standard of surgical training which is competency based. Further, the 2025 orthopaedic training intake has reached critical mass for the first time with over 30% of trainees identifying as female.^111^ However, as seen in law and the veterinary profession, reaching gender equity does not automatically resolve deeper systemic issues, such as gender discrimination, unequal access to leadership roles and persistent workplaces culture challenges.^112^, ^113^ This highlights the need for continued efforts to address these underlying barriers even as gender parity is approached in training.

### Strengths and limitations

This scoping review has strengths and limitations. The search strategy was comprehensive but could have missed eligible articles. This is unlikely to have altered the findings of the review due to the large number of included studies spanning study designs and sample sizes. The findings from this review may not apply to low‐ and middle‐income countries, as studies from these settings were excluded from the review. Additionally, included studies predominantly originated from North America. The data on the Australian climate was limited to a single article. Notably no studies from East Asia or Nordic countries met the inclusion criteria, limiting insights into gender dynamics in those contexts. This geographical gap is unlikely to have significantly altered the overall findings due to the large number of included studies spanning diverse cultural and religious backgrounds. However, the lack of representation from these regions limits the generalisability of the findings. Additionally, most included studies were cross‐sectional surveys, which inherently limits the ability to establish causality and is subject to non‐response bias, as evidenced by the MMAT quality analysis. The use of self‐reported data in many studies introduces the possibility of response bias, as participants might underreport or overreport certain experiences due to social desirability or recall bias. Furthermore, the varied methodological quality of the included studies, as assessed by the MMAT, indicates that some findings may be based on lower‐quality evidence. Despite these limitations, the consistent themes identified across studies suggest robustness in the findings. Lastly, the dynamic and evolving nature of gender diversity issues means that the findings of this review may need regular updating to remain relevant.

### Evidence gaps and future research

This scoping review confirms that female orthopaedic surgeons worldwide encounter various barriers and facilitators during their training and practice. However, there is a significant gap in research on the effectiveness of interventions aimed at addressing these issues. Future research should focus on evaluating specific interventions to determine their impact on achieving critical mass and enhancing patient care. Additionally, most existing studies originate from North America, highlighting the need for more research in low‐income regions, Australasia, Scandinavia, and other underrepresented high‐income countries. This research could provide a comprehensive understanding of the global context and guide the implementation of effective strategies and policies to promote gender equity, ultimately creating a more inclusive and diverse orthopaedic workforce.

### Conclusions

Achieving gender equity in orthopaedic surgery requires concerted efforts at multiple levels. This scoping review highlights the critical areas where interventions can be most effectively focused and emphasizes the breadth of areas organizations can focus on to achieve gender parity. By addressing the identified barriers and amplifying the facilitators, the orthopaedic community can move towards a more inclusive and diverse workforce, ultimately enhancing the quality of care provided to patients.

## Author contributions

**Clara Freeman:** Conceptualization; data curation; formal analysis; investigation; methodology; project administration; writing – original draft. **Rebecca Evans:** Supervision; writing – review and editing. **Natalie Drever:** Formal analysis; methodology; writing – review and editing. **Jordy White:** Investigation; writing – review and editing. **Sarah Larkins:** Supervision; writing – review and editing. **Christopher Morrey:** Supervision; writing – review and editing.

## Conflict of interest

None declared.

## Supporting information


**Table S1.** PICO inclusion and exclusion criteria.

**Table S2.** Keyword search updated 14 July 2024.

**Table S3.** Mixed methods appraisal tool.^24^

**Table S4.** Excluded studies during full text screening with reason.

**Table S5.** characteristics of included studies.

**Table S6.** Barriers and facilitators for female practitioners in orthopaedic surgery mapped to the socio‐ecological model.

**Table S7.** Quality assessment of included studies using the MMAT.^24^

**Table S8.** Quality assessment of included studies using the mixed methods appraisal tool.^24^

## References

[1] #GenderEqualOlympics: Celebrating full gender parity on the field of play at Paris 2024. International Olympics Committee. [Cited 19 Aug 2024.] Available from URL: https://olympics.com/ioc/news/genderequalolympics‐celebrating‐full‐gender‐parity‐on‐the‐field‐of‐play‐at‐paris‐2024.

[2] Gomez LE , Bernet P . Diversity improves performance and outcomes. J. Natl. Med. Assoc. 2019; 111: 383–392. 30765101 10.1016/j.jnma.2019.01.006

[3] Arias C , Bytyqui D , Chokotho L *et al*. Diversity in orthopaedics and traumatology: a global perspective. EFORT Open Rev. 2020; 5: 743–752. 33204518 10.1302/2058-5241.5.200022 PMC7608568

[4] Kanter RM . Some effects of proportions on group life: skewed sex ratios and responses to token women. Am. J. Sociol. 1977; 82: 965–990.

[5] Joecks J , Pull K , Vetter K . Gender diversity in the boardroom and firm performance: what exactly constitutes a "critical mass?". J. Bus. Ethics 2013; 118: 61–72.

[6] Acuña AJ , Sato EH , Jella TK *et al*. How long will it take to reach gender parity in orthopaedic surgery in the United States? An analysis of the National Provider Identifier Registry. Clin. Orthop. Relat. Res. 2021; 479: 1179–1189. 33871403 10.1097/CORR.0000000000001724 PMC8133193

[7] Newman TH , Parry MG , Zakeri R *et al*. Gender diversity in UK surgical specialties: a national observational study. BMJ Open 2022; 12: e055516. 10.1136/bmjopen-2021-055516 PMC8968535 35314455

[8] Morimoto T , Kobayashi T , Yamauchi K *et al*. How long will it take to reach the gender diversity goal for orthopaedics in Japan? J. Orthop. Sci. 2024; 29: 1140–1144. 37308331 10.1016/j.jos.2023.05.011

[9] Graham V , Arora B . Women in surgery: trends in nine surgical specialties. ANZ J. Surg. 2023; 93: 2344–2349. 37458242 10.1111/ans.18600

[10] Rama E , Ekhtiari S , Thevendran G , Green J , Weber K , Khanduja V . Overcoming the barriers to diversity in Orthopaedic surgery: a global perspective. J. Bone Jt. Surg. Am. 2023; 105: 1910–1919. 10.2106/JBJS.23.00238 37639495

[11] Green JA , Chye VP , Hiemstra LA *et al*. Diversity: women in orthopaedic surgery: a perspective from the International Orthopaedic Diversity Alliance. J. Orthop. Trauma 2020; 8: 44–51.

[12] Clark M , Kerslake S , Bøe B , Hiemstra LA . Being a woman and an orthopaedic surgeon—a primer on the challenges we face. J. ISAKOS 2024; 9: 449–456. 38777119 10.1016/j.jisako.2024.05.008

[13] Lewis VO , Scherl SA , O'Connor MI . Women in Orthopaedics—way behind the number curve. J. Bone Jt. Surg. Am. 2012; 94: e30. 10.2106/JBJS.J.01408 22398744

[14] Pechlivanidou E , Antonopoulos I , Margariti RE . Gender equality challenges in orthopaedic surgery: a systematic review. Int. Orthop. 2023; 47: 2143–2171. 37433883 10.1007/s00264-023-05876-w

[15] Enson J , Malik‐Tabassum K , Faria A , Faria G , Gill K , Rogers B . The impact of mentoring in trauma and orthopaedic training: a systematic review. Ann. R. Coll. Surg. Engl. 2022; 104: 400–408. 35446153 10.1308/rcsann.2021.0330 PMC9157945

[16] Morrison LJ , Abbott AG , Mack Z , Schneider P , Hiemstra LA . What are the challenges related to family planning, pregnancy, and parenthood faced by women in orthopaedic surgery? A systematic review. Clin. Orthop. Relat. Res. 2023; 481: 1307–1318. 36853855 10.1097/CORR.0000000000002564 PMC10263240

[17] O'Connor MI . Medical school experiences shape women students' interest in orthopaedic surgery. Clin. Orthop. Relat. Res. 2016; 474: 1967–1972. 27084717 10.1007/s11999-016-4830-3 PMC4965370

[18] Gianakos AL , Freischlag JA , Mercurio AM *et al*. Bullying, discrimination, harassment, sexual harassment, and the fear of retaliation during surgical residency training: a systematic review. World J. Surg. 2022; 46: 1587–1599. 35006329 10.1007/s00268-021-06432-6

[19] Daudt HM , Van Mossel C , Scott SJ . Enhancing the scoping study methodology: a large, inter‐professional team's experience with Arksey and O'Malley's framework. BMC Med. Res. Methodol. 2013; 13: 1–9. 23522333 10.1186/1471-2288-13-48 PMC3614526

[20] Kilanowski JF . Breadth of the socio‐ecological model. J. Agromedicine 2017; 22: 295–297. 28742433 10.1080/1059924X.2017.1358971

[21] Tricco AC , Lillie E , Zarin W *et al*. PRISMA extension for scoping reviews (PRISMA‐ScR): checklist and explanation. Ann. Intern. Med. 2018; 169: 467–473. 30178033 10.7326/M18-0850

[22] Eriksen MB , Frandsen TF . The impact of patient, intervention, comparison, outcome (PICO) as a search strategy tool on literature search quality: a systematic review. J. Med. Library Assoc. JMLA. 2018; 106: 420–431. 10.5195/jmla.2018.345 PMC6148624 30271283

[23] Covidence systematic review software . Veritas Health Innovation. [Cited 3 Sept 2024.] Available from URL: www.covidence.org.

[24] Hong QN , Fàbregues S , Bartlett G *et al*. The mixed methods appraisal tool (MMAT) version 2018 for information professionals and researchers. Educ. inform. 2018; 34: 285–291.

[25] Leinweber J , Creedy DK , Rowe H , Gamble J . A socioecological model of posttraumatic stress among Australian midwives. Midwifery 2017; 45: 7–13. 27960122 10.1016/j.midw.2016.12.001

[26] Hennein R , Lowe S . A hybrid inductive‐abductive analysis of health workers' experiences and wellbeing during the COVID‐19 pandemic in the United States. PLoS One 2020; 15: e0240646. 33104711 10.1371/journal.pone.0240646 PMC7588050

[27] Chen Y , Zhang R , Lou Y , Li W , Yang H . Facilitators and barriers to the delivery of palliative care to patients with Parkinson's disease: a qualitative study of the perceptions and experiences of stakeholders using the socio‐ecological model. BMC Health Serv. Res. 2023; 23: 215. 36879235 10.1186/s12913-023-09203-2 PMC9990289

[28] Hiemstra LA , Kerslake S , Clark M , Temple‐Oberle C , Boynton E . Experiences of Canadian female Orthopaedic surgeons in the workplace: defining the barriers to gender equity. J. Bone Joint Surg. Am. 2022; 104: 1455–1461. 35594484 10.2106/JBJS.21.01462

[29] Thiart M , O'Connor M , Müller J , Holland N , Bantjes J . Operating in the margins: Women's lived experience of training and working in orthopaedic surgery in South Africa. Qual. Res. Med. Healthc. 2023; 7: 20–31. 10.4081/qrmh.2023.10902 PMC10336873 37441128

[30] Tosi LL , Mankin HJ . Ensuring the success of women in academic orthopaedics. Clin. Orthop. Relat. Res. 1998; 356: 254–263. 10.1097/00003086-199811000-00034 9917692

[31] Brady JM , Bray A , Kim P *et al*. Female residents give themselves lower scores than male colleagues and faculty evaluators on ACGME milestones. J. Surg. Educ. 2021; 78: 1305–1311. 33349566 10.1016/j.jsurg.2020.12.003

[32] Hill JF , Johnson AH , Cannada L . A profile of female academic orthopaedic surgeons. Curr. Orthopaed. Pract. 2013; 24: 636–640.

[33] Dupley L , Hossain S , Ghosh S . Performance anxiety amongst trauma and orthopaedic surgical trainees. Surgeon (Elsevier Science). 2020; 18: e33–e38. 10.1016/j.surge.2020.06.002 32653398

[34] Sargent MC , Sotile W , Sotile MO *et al*. Managing stress in the orthopaedic family: avoiding burnout, achieving resilience. J. Bone Jt. Surg. Am. 2011; 93: e40. 10.2106/JBJS.J.01252 21508275

[35] Hiemstra LA , Kerslake S , Fritz J‐A *et al*. Rates of burnout in female orthopaedic surgeons correlate with barriers to gender equity. JBJS 2023; 105: 849–854. 10.2106/JBJS.22.01319 37083849

[36] Rodarte P , Kammire MS , Israel H , Poon SC , Cannada LK . The other side of conflict: examining the challenges of female orthopaedic surgeons in the workplace. Am. J. Surg. 2023; 225: 46–52. 36243560 10.1016/j.amjsurg.2022.09.027

[37] Alshammari AN , Shafiq MO , Altayeb MA , Khaja AF , Ghabban KM , Khoshhal KI . Gulf cooperation council female residents in orthopedics: influences, barriers, and mental pressures: a cross‐sectional study. J. Musculoskeletal Surg. Res. 2018; 2: 51.

[38] Cafruni VM , Cabas Geat A , Labella JF *et al*. Women's role in orthopedic training programs: what proportion do they represent today? Rev Fac Cien Med Univ Nac Cordoba 2022; 79: 15–18. 35312261 10.31053/1853.0605.v79.n1.28184 PMC9004294

[39] Higgins MJ , Kale NN , Brown SM , Mulcahey MK . Taking family call: understanding how Orthopaedic surgeons manage home, family, and life responsibilities. J. Am. Acad. Orthopaed. Surg. 2021; 29: e31–e40. 10.5435/JAAOS-D-20-00182 32568993

[40] Ponzio DY , Bell C , Stavrakis A *et al*. Discrepancies in work‐family integration between female and male Orthopaedic surgeons. J. Bone Jt. Surg. Am Vol. 2022; 104: 465–472. 10.2106/JBJS.21.00345 34851322

[41] Rohde R , Wolf J , Adams J , Rohde RS , Wolf JM , Adams JE . Where are the women in orthopaedic surgery? Clin. Orthopaed. Related Res. 2016; 474: 1950–1956. 10.1007/s11999-016-4827-y PMC4965367 27090259

[42] Downie S , Cherry J , Dunn J *et al*. The role of gender in operative autonomy in orthopaedic surgical trainees (GOAST): a national collaborative project. Bone Jt. J. 2023; 105: 821–832. 10.1302/0301-620X.105B7.BJJ-2023-0132.R2 37399113

[43] Mulcahey MK , Nemeth C , Trojan JD , O'Connor MI . The perception of pregnancy and parenthood among female orthopaedic surgery residents. J. Am. Acad. Orthopaed. Surg. 2019; 27: 527–532. 10.5435/JAAOS-D-18-00216 30499893

[44] Wynn M , Lawler E , Schippers S , Hajewski T , Weldin E , Campion H . Pregnancy during orthopaedic surgery residency: the Iowa experience. Iowa Orthop. J. 2022; 42: 11–14. 35821958 PMC9210436

[45] Reid DBC , Shah KN , Lama CJ , Kosinski LR , Daniels AH , Eberson CP . Parenthood among orthopedic surgery residents: assessment of resident and program director perceptions on training. Orthopedics 2021; 44: 98–104. 33561867 10.3928/01477447-20210201-08

[46] Ruse S , Bergman R , Crawford E . Pregnancy in orthopaedic residents: peripartum barriers identified. JBJS Open Access 2022; 7: e22. 10.2106/JBJS.OA.22.00098. PMC9788970 36601291

[47] Nemeth C , Roll E , Mulcahey MK . Program directors' perception of pregnancy and parenthood in orthopedic surgery residency. Orthopedics 2020; 43: e109–e113. 31841611 10.3928/01477447-20191212-02

[48] Hill JF , Yule A , Zurakowski D , Day CS . Residents' perceptions of sex diversity in orthopaedic surgery. J. Bone Jt. Surg. Am. Vol. 2013; 95: e1441–e1446. 10.2106/JBJS.L.00666 24088979

[49] Jurenovich KM , Cannada LK . Women in orthopedics and their fellowship choice: what influenced their specialty choice? Iowa Orthop. J. 2020; 40: 13–17. 32742203 PMC7368530

[50] Lurie B , Albanese J , Allenback G , Elliott I , Nelson K . Small glove size and female gender are associated with greater reported difficulty using orthopaedic instruments among residents. JBJS Open Access 2024; 9: e23. 10.2106/JBJS.OA.23.00151. PMC11108343 38779173

[51] Tan R , Bond E , Muir D . Perceived barriers to a career in orthopaedic surgery for women: a comparison between orthopaedic and general surgery. ANZ J. Surg. 2021; 91: 1650–1651. 34506058 10.1111/ans.17112

[52] Xu AL , Humbyrd CJ , De Mattos CBR , LaPorte D . The importance of perceived barriers to women entering and advancing in orthopaedic surgery in the US and beyond. World J. Surg. 2023; 47: 3051–3059. 37735223 10.1007/s00268-023-07165-4

[53] Balch Samora J , Denning J , Haralabatos S , Luong M , Poon S . Do women experience microaggressions in orthopaedic surgery? Current state and future directions from a survey of women orthopaedists. Curr. Orthopaed. Pract. 2020; 31: 503–507.

[54] Sobel AD , Lavorgna TR , Ames SE , Templeton KJ , Mulcahey MK . Interpersonal interactions and biases in orthopaedic surgery residency: do experiences differ based on gender? Clin. Orthop. Relat. Res. 2023; 481: 369–378. 36668700 10.1097/CORR.0000000000002457 PMC9831198

[55] Alhammadi NA , Jabbar IA , Alahmari SA *et al*. Gender‐related microaggressions in orthopedic surgery: a comprehensive survey of women orthopedists and implications for Progress, Saudi Arabia. J. Healthc. Lead. 2024; 16: 29–37. 10.2147/JHL.S437083 PMC10785692 38223496

[56] Samora JB , Van Heest A , Weber K , Ross W , Huff T , Carter C . Harassment, discrimination, and bullying in orthopaedics: a work environment and culture survey. JAAOS‐J. Am. Acad. Orthopaed. Surg. 2020; 28: e1097–e1104. 10.5435/JAAOS-D-19-00822 32187075

[57] Giglio V , Schneider P , Bond Z *et al*. Prevalence of gender‐based and sexual harassment within orthopedic surgery in Canada. Can. J. Surg. 2022; 65: E45–E51. 35086850 10.1503/cjs.013120 PMC8802889

[58] Whicker E , Williams C , Kirchner G , Khalsa A , Mulcahey MK . What proportion of women orthopaedic surgeons report having been sexually harassed during residency training? A survey study. Clin. Orthop. Relat. Res. 2020; 478: 2598–2606. 32956144 10.1097/CORR.0000000000001454 PMC7571927

[59] Bucknall V , Pynsent PB , Bucknall V , Pynsent PB . Sex and the orthopaedic surgeon: a survey of patient, medical student and male orthopaedic surgeon attitudes towards female orthopaedic surgeons. Surgeon (Edinburgh University Press). 2009; 7: 89–95. 10.1016/s1479-666x(09)80023-1 19408801

[60] Huntington WP , Haines N , Patt JC . What factors influence applicants' rankings of orthopaedic surgery residency programs in the National Resident Matching Program? Clin. Orthopaed. Related Res. 2014; 472: 2859–2866. 10.1007/s11999-014-3692-9 PMC4117903 24898527

[61] Yonai Y , Masarwa R , Steinfeld Y , Ben Natan M , Berkovich Y . Israeli female Physicians' motives for choosing orthopedics as their specialty choice. World J. Surg. 2023; 47: 1364–1370. 36894699 10.1007/s00268-023-06953-2

[62] Meert C , Manon J , Cornu O . Female representation in orthopedic surgery: where do we stand in Belgium ? Acta Orthop. Belg. 2023; 89: 671–677. 38205759 10.52628/89.4.12184

[63] Ponce B , Gruenberger E , McGwin G , Samora J , Patt J . Workplace violence in orthopaedic surgery: a survey of academy of orthopaedic surgeons membership. J. Am. Acad. Orthop. Surg. 2024; 32: e359–e367. 38442420 10.5435/JAAOS-D-23-00596

[64] London DA , Calfee RP , Boyer MI . Impact of a musculoskeletal clerkship on orthopedic surgery applicant diversity. Am. J. Orthop. (Belle Mead N.J.) 2016; 45: E347–E351. 27737294

[65] Goss ML , McNutt SE , Hallan DR , Bible JE . Factors in Orthopaedic residency decision‐making for female applicants: a cross‐sectional study. J. Am. Acad. Orthopaed. Surg. 2020; 28: 1055–1060. 10.5435/JAAOS-D-20-00026 32355053

[66] Okike K , Phillips DP , Swart E , O'Connor MI . Orthopaedic faculty and resident sex diversity are associated with the Orthopaedic residency application rate of female medical students. J. Bone Jt. Surg. Am. Vol. 2019; 101: e56. 10.2106/JBJS.18.00320 31220032

[67] Sabesan VJ , Lavin A , Lama G *et al*. The sex or race of program directors may not play a significant role in impacting diversity among Orthopaedic surgery residents. Arthrosc. J. Arthrosc. Relat. Surg. 2024. in press. 10.1016/j.arthro.2024.03.036. 38593927

[68] Vivekanantha P , Dao A , Hiemstra L *et al*. Gender representation in major orthopaedic surgery meetings: a quantitative analysis. JBJS Open Access 2023; 8: e23. 10.2106/JBJS.OA.23.00067 PMC10619889 37920560

[69] Bauer JM , Holt GE . National orthopedic residency attrition: who is At risk? J. Surg. Educ. 2016; 73: 852–857. 27216301 10.1016/j.jsurg.2016.03.010

[70] Gerull KM , Pérez M , Cipriano CA , Jeffe DB . Orthopaedic surgery attrition before board certification: a national‐cohort study of US MD graduates in orthopaedic surgery residency programs. JBJS Open Access 2024; 9: e23. 10.2106/JBJS.OA.23.00175. PMC11101223 38770154

[71] Haruno LS , Chen X , Metzger M *et al*. Racial and sex disparities in resident attrition in orthopaedic surgery. JBJS Open Access 2023; 8: e22. 10.2106/JBJS.OA.22.00148. PMC10284323 37351087

[72] Walker JL , Janssen H , Hubbard D . Gender differences in attrition from orthopaedic surgery residency. J. Am. Med. Womens Assoc. (1972) 1993; 48: 182–193. 8263275

[73] Brook EM , Hu CH , Li X , Smith EL , Matzkin EG . The influence of mentors in orthopedic surgery. Orthopedics 2020; 43: e37–e42. 31770444 10.3928/01477447-20191122-02

[74] Gerull KM , Kim DJ , Cogsil T , Rhea L , Cipriano C . Are women proportionately represented as speakers at orthopaedic surgery annual meetings? A cross‐sectional analysis. Clin. Orthop. Relat. Res. 2020; 478: 2729–2740. 32667757 10.1097/CORR.0000000000001359 PMC7899418

[75] Nwosu C , Wittstein JR , Erickson MM *et al*. Representation of female speakers at the American Academy of Orthopaedic surgeons annual meetings over time. J. Am. Acad. Orthop. Surg. 2023; 31: 283–291. 36727899 10.5435/JAAOS-D-22-00615

[76] Hoof MA , Sommi C , Meyer LE , Bird ML , Brown SM , Mulcahey MK . Gender‐related differences in research productivity, position, and advancement among academic orthopaedic faculty within the United States. J. Am. Acad. Orthop. Surg. 2020; 28: 893–899. 32049692 10.5435/JAAOS-D-19-00408

[77] Ramos T , Daban R , Kale N *et al*. Women in leadership in state and regional Orthopaedic societies. J. Am. Acad. Orthop. Surg. Glob. Res. 2022; 6: e21. 10.5435/JAAOSGlobal-D-21-00317. PMC8990971 35389899

[78] Sobel AD , Cox RM , Ashinsky B , Eberson CP , Mulcahey MK . Analysis of factors related to the sex diversity of Orthopaedic residency programs in the United States. J. Bone Jt. Surg. Am. Vol. 2018; 100: 1–6. 10.2106/JBJS.17.01202 PMC6818998 29870453

[79] Gerull KM , Holten A , Rhea L , Cipriano C . Is the distribution of awards gender‐balanced in orthopaedic surgery societies? Clin. Orthop. Relat. Res. 2021; 479: 33–43. 32555007 10.1097/CORR.0000000000001364 PMC7899733

[80] Buerba RA , Arshi A , Greenberg DC , SooHoo NF . The role of gender, academic affiliation, and subspecialty in relation to industry payments to orthopaedic surgeons. J. Natl. Med. Assoc. 2020; 112: 82–90. 31685219 10.1016/j.jnma.2019.09.004

[81] Avila A , Sato EH , Acuna AJ , Vasavada K , Kamath AF . Gender differences in reimbursement among orthopaedic surgeons: a cross‐sectional analysis of Medicare claims. J. Am. Acad. Orthop. Surg. 2023; 31: e570–e578. 37253194 10.5435/JAAOS-D-22-00823

[82] Beebe KS , Krell ES , Rynecki ND , Ippolito JA . The effect of sex on orthopaedic surgeon income. J. Bone Joint Surg. Am. 2019; 101: e87. 31483407 10.2106/JBJS.18.01247

[83] Dossa F , Simpson AN , Sutradhar R *et al*. Sex‐based disparities in the hourly earnings of surgeons in the fee‐for‐service system in Ontario, Canada. JAMA Surg. 2019; 154: 1134–1142. 31577348 10.1001/jamasurg.2019.3769 PMC6777399

[84] Forrester LA , Seo LJ , Gonzalez LJ , Zhao C , Friedlander S , Chu A . Men receive three times more industry payments than women academic orthopaedic surgeons, even after controlling for confounding variables. Clin. Orthop. Relat. Res. 2020; 478: 1593–1599. 31977436 10.1097/CORR.0000000000001132 PMC7310494

[85] Jena AB , Olenski AR , Blumenthal DM . Sex differences in physician salary in US public medical schools. JAMA Intern. Med. 2016; 176: 1294–1304. 27400435 10.1001/jamainternmed.2016.3284 PMC5558151

[86] Bohl DD , Iantorno SE , Kogan M . Inappropriate questions asked of female Orthopaedic surgery applicants from 1971 to 2015: a cross‐sectional study. J. Am. Acad. Orthopaed. Surg. 2019; 27: 519–526. 10.5435/JAAOS-D-17-00868 30399030

[87] Theiss LM , Prather JC , Porterfield JR *et al*. Prevalence, bias, and rank list impact of illegal questions in surgical specialty residency interviews. J. Surg. Educ. 2022; 79: 69–76. 34400121 10.1016/j.jsurg.2021.07.015

[88] Girgis MY , Qazi S , Patel A , Yu D , Lu X , Sewards J . Gender and racial bias in letters of recommendation for orthopedic surgery residency positions. J. Surg. Educ. 2023; 80: 127–134. 36151044 10.1016/j.jsurg.2022.08.021

[89] Kobayashi AN , Sterling RS , Tackett SA , Chee BW , Laporte DM , Humbyrd CJ . Are there gender‐based differences in language in letters of recommendation to an orthopaedic surgery residency program? Clin. Orthop. Relat. Res. 2020; 478: 1400–1408. 31794493 10.1097/CORR.0000000000001053 PMC7310286

[90] Powers A , Gerull KM , Rothman R , Klein SA , Wright RW , Dy CJ . Race‐ and gender‐based differences in descriptions of applicants in the letters of recommendation for orthopaedic surgery residency. JBJS Open Access 2020; 5: e20. 10.2106/JBJS.OA.20.00023. PMC7386551 32803104

[91] Incoll IW , Atkin J , Frank JR , Vrancic S , Khorshid O . Gender associations with selection into Australian orthopaedic surgical training: 2007‐2019. ANZ J. Surg. 2021; 91: 2757–2766. 34723445 10.1111/ans.17320

[92] Poon S , Nellans K , Crabb RAL *et al*. Academic metrics do not explain the underrepresentation of women in Orthopaedic training programs. J. Bone Jt. Surg. Am. Vol. 2019; 101: e32. 10.2106/JBJS.17.01372 30994596

[93] Webber CRJ , Davie R , Herzwurm Z , Whitehead J , Pare DW , Homlar KC . Is there unconscious bias in the orthopaedic residency interview selection process? J. Surg. Educ. 2022; 79: 1055–1062. 35241397 10.1016/j.jsurg.2022.02.003

[94] Peck CJ , Schmidt SJ , Latimore DA , O'Connor MI . Chair versus chairman: does orthopaedics use the gendered term more than other specialties? Clin. Orthop. Relat. Res. 2020; 478: 1583–1589. 31567285 10.1097/CORR.0000000000000964 PMC7310475

[95] Nguyen CV , Luong M , Weiss JM , Hardesty C , Karamitopoulos M , Poon S . The cost of maternity leave for the orthopaedic surgeon. J. Am. Acad. Orthop. Surg. 2020; 28: e1001–e1005. 32079849 10.5435/JAAOS-D-19-00337

[96] Wynn M , Caldwell L , Kowalski H , Lawler E . Identifying barriers: current breastfeeding policy in orthopedic surgery residency. Iowa Orthop. J. 2021; 41: 5–9. 34552396 PMC8259184

[97] DelPrete CR , Gianakos A , LaPorte D , Ierulli VK , Mulcahey MK . Perception and usage of social media among women in Orthopaedics. J. Am. Acad. Orthopaed. Surg. Global Res. rev. 2023; 7: e23. 10.5435/JAAOSGlobal-D-23-00100. PMC10656082 37973034

[98] Harbold D , Dearolf L , Buckley J , Lattanza L . The Perry initiative's impact on gender diversity within orthopedic education. Curr. Rev. Musculoskelet. Med. 2021; 14: 429–433. 34626321 10.1007/s12178-021-09717-4 PMC8501316

[99] Mason B , Ross W , Ortega G *et al*. Can a strategic pipeline initiative increase the number of women and underrepresented minorities in orthopaedic surgery? Clin. Orthopaed. Related Res. 2016; 474: 1979–1985. 10.1007/s11999-016-4846-8 PMC4965371 27113596

[100] Kroin E , Garbarski D , Shimomura A , Romano J , Schiff A , Wu K . Gender differences in program factors important to applicants when evaluating orthopaedic surgery residency programs. J. Grad. Med. Educ. 2019; 11: 565–569. 31636827 10.4300/JGME-D-18-01078.1 PMC6795318

[101] Julian KR , Anand M , Sobel AD , Mulcahey MK , Wong SE . A 5‐year update and comparison of factors related to the sex diversity of orthopaedic residency programs in the United States. JBJS Open Access 2023; 8: e22. 10.2106/JBJS.OA.22.00116. PMC9990829 36896147

[102] Saxena S , Cannada LK , Weiss JM . Does the proportion of women in orthopaedic leadership roles reflect the gender composition of specialty societies? Clin. Orthop. Relat. Res. 2020; 478: 1572–1579. 31180910 10.1097/CORR.0000000000000823 PMC7310307

[103] Levy KH , Gupta A , Murdock CJ *et al*. Effect of faculty diversity on minority student populations matching into orthopaedic surgery residency programs. JBJS Open Access 2023; 8: e22. 10.2106/JBJS.OA.22.00117 PMC9820786 36698980

[104] Munger AM , Heckmann N , McKnight B , Dusch MN , Hatch GF 3rd , Omid R . Revisiting the gender gap in Orthopaedic surgery: investigating the relationship between Orthopaedic surgery female faculty and female residency applicants. J. Am. Acad. Orthopaed. Surg. 2018; 26: 295–300. 10.5435/JAAOS-D-17-00686 30278014

[105] Stevens CR , Merk K , Ierulli VK , Mulcahey MK . Analysis of social media posts that promote women surgeons. J. Surg. Educ. 2023; 80: 682–688. 36872167 10.1016/j.jsurg.2023.02.002

[106] Wang KY , Puvanesarajah V , Suresh KV *et al*. Social media presence is associated with diversity and application volume for orthopedic surgery residency programs. Orthopedics 2023; 46: 47–53. 36314878 10.3928/01477447-20221024-01

[107] AMA Position Statement on Medical parents and prevocational and vocational training Australian Medical Association ; 2024, https://www.ama.com.au/media/support-needed-parents-medical-training.

[108] Are we there yet? Australian Medical Association New South Wales; 2024. https://www.amansw.com.au/are-we-there-yet/.

[109] Becoming an orthopaedic surgeon . Australian Orthopaedic Association; 2024, https://aoa.org.au/orthopaedic-training/becoming-an-orthopaedic-surgeon.

[110] Turban S , Wu D , Zhang L . When gender diversity makes firms more productive. Harv. Bus. Rev. 2019; 11: 17.

[111] Successful applicants for the 2026 intake . Sydney, Australia: Australian Orthopaedic Association. [Cited 20 Aug 2024.] Available from URL: 2025. https://aoa.org.au/orthopaedic-training/selection-into-training.

[112] Thornton M . Who cares? The conundrum for gender equality in legal practice. UNSWLJ 2020; 43: 1473.

[113] Begeny CT , Ryan MK , Moss‐Racusin CA , Ravetz G . In some professions, women have become well represented, yet gender bias persists—perpetuated by those who think it is not happening. Sci. Adv. 2020; 6: eaba7814. 32637616 10.1126/sciadv.aba7814 PMC7319752

