# Supplementary material for: Barriers and facilitators for female practitioners in orthopaedic training and practice: a scoping review
Source: ANZ J Surg. 2025 Jan 3;95(4):647–57. doi: 10.1111/ans.19334 (PMC11982664; doi:10.1111/ans.19334)
Supplement: Supplementary file 1 — Table S1. PICO inclusion and exclusion criteria. [file ANS-95-647-s002.docx]

**TABLE S1:** PICO inclusion and exclusion criteria

|  | INCLUSION | EXCLUSION |
| --- | --- | --- |
| POPULATION | Orthopaedic surgeons – trainees/ registrars, consultants  High income countries | Orthopaedic subspecialty comparisons  Medical students, interns, unaccredited trainees |
| INTERVENTION/ EXPOSURE |  | Orthopaedic procedures |
| COMPARATOR/CONTEXT | Female Practitioners |  |
| OUTCOME | Orthopaedic surgery  Training programs |  |
| STUDY CHARACTERISTICS | Original research | Reviews  Case studies  Opinion pieces  Grey literature |
| OTHER | Barriers and facilitators discussed | Articles on statistical demographics of surgical groups without discussion of reasoning why or implications |
