# Supplementary material for: Barriers and facilitators for female practitioners in orthopaedic training and practice: a scoping review
Source: ANZ J Surg. 2025 Jan 3;95(4):647–57. doi: 10.1111/ans.19334 (PMC11982664; doi:10.1111/ans.19334)
Supplement: Supplementary file 2 — Table S2. Keyword search updated 14 July 2024. [file ANS-95-647-s008.docx]

**TABLE S2:** Keyword search updated 14 July 2024

**MEDLINE (OVID)**

| NUMBER | SEARCH | RESULT |
| --- | --- | --- |
| 1 | Female/ | 9834003 |
| 2 | exp Women/ | 46085 |
| 3 | 1 OR 2 | 9841488 |
| 4 | exp Surgeons/ | 18894 |
| 5 | exp Education, Medical, Graduate/ | 84748 |
| 6 | Physicians/ or physicians, women/ | 111865 |
| 7 | 4 OR 5 OR 6 | 207525 |
| 8 | Orthopedics/ | 24797 |
| 9 | exp Orthopedic surgeons/ | 1483 |
| 10 | 8 OR 9 | 25842 |
| 11 | 3 AND 7 AND 10 | 1079 |

**CINAHL**

| NUMBER | SEARCH | RESULT |
| --- | --- | --- |
| S1 | (MH “Female”) OR (MH “Women+”) | 2,289,615 |
| S2 | (MH “Physicians”) OR (MH “Physicians, Women”) | 68,240 |
| S3 | MH “Surgeons” | 13,246 |
| S4 | (MH “Education, Medical+”) OR (MH “Internship and residency+”) OR (MH “Interns and residents”) | 67,513 |
| S5 | S2 OR S3 OR S4 | 142,624 |
| S6 | MH “Orthopedic Surgery” | 19,520 |
| S7 | MH “Orthopedics” | 12,572 |
| S8 | S6 OR S7 | 30,063 |
| S9 | S1 AND S5 AND S8 | 525 |

**EMCARE (OVID)**

| NUMBER | SEARCH | RESULT |
| --- | --- | --- |
| 1 | exp Female/ | 1987972 |
| 2 | Physician/ OR female physician/ | 163749 |
| 3 | Surgeon/ | 33984 |
| 4 | Medical education/ or medical school/ or residency education/ or surgical training/ | 88914 |
| 5 | 2 OR 3 OR 4 | 268392 |
| 6 | exp Orthopedics/ | 9539 |
| 7 | Orthopedic surgery/ | 11591 |
| 8 | 6 OR 7 | 20438 |
| 9 | 1 AND 5 AND 8 | 624 |

**EMBASE**

| NUMBER | SEARCH | RESULT |
| --- | --- | --- |
| 1 | 'female':ab,kw,ti | 1,475,444 |
| 2 | 'physician':ab,kw,ti | 351,639 |
| 3 | 'surgeon':ab,kw,ti | 186,051 |
| 4 | 'medical education':ab,kw,ti | 78,772 |
| 5 | 'medical school':ab,kw,ti | 39,857 |
| 6 | 'residency education':ab,kw,ti | 1,523 |
| 7 | 'surgical training':ab,kw,ti | 9,804 |
| 8 | #2 OR #3 OR #4 OR #5 OR #6 OR #7 | 638,027 |
| 9 | 'orthopedics':ab,kw,ti | 16,018 |
| 10 | 'orthopedic surgeon':ab,kw,ti | 3,038 |
| 11 | 'orthopedic surgery':ab,kw,ti | 14,024 |
| 12 | #9 OR #10 OR #11 | 31,955 |
| 13 | #1 AND #8 AND #12 | 410 |

**SCOPUS**

| NUMBER | SEARCH | RESULT |
| --- | --- | --- |
| S1 | TITLE-ABS-KEY ( "women*" OR "woman" OR "girl" OR "girls" OR "female" ) | 12,950,180 |
| S2 | TITLE-ABS-KEY ( "surgeon" OR "surgeons" OR "surgical expert" OR "operating physician" OR "physician" OR "physicians" OR "female physician" OR "wom* physician" OR "medical practitioner" OR "education, graduate medical" OR "education, medical, graduate" OR "graduate medical education" OR "medical education graduate" OR "internship and residency" OR "internship" OR "house staff" OR "internship, medical" "medical internship" OR "medical internships" OR "medical residencies" OR "residencies" OR "residencies, medical" OR "residency" OR "residency, medical" OR "residency and internship" OR "staff, house" OR "surgical registrar" ) | 69,696 |
| S3 | TITLE-ABS-KEY ( "orthopedic surgeon" OR "orthopedic surgeons" OR "orthopedist" OR "orthopedists" OR "surgeon, orthopedic" OR "surgeons, orthopedic" OR "orthopedics" OR "orthopaedic surgeon" OR "orthopaedic surgeons" OR "orthopaedist" OR "orthopaedists" OR "surgeon, orthopaedic" OR "surgeons, orthopaedic" OR "orthopaedics" ) | 230,902 |
| S4 | S1 AND S2 AND S3  TITLE-ABS-KEY ( "women" OR "woman" OR "girl" OR "girls" OR "women groups" OR "women's groups" OR "female" ) AND TITLE-ABS-KEY ( "surgeon" OR "surgeons" OR "surgical expert" OR "operating physician" OR "physician" OR "physicians" OR "female physician" OR "wom* physician" OR "medical practitioner" OR "education, graduate medical" OR "education, medical, graduate" OR "graduate medical education" OR "medical education graduate" OR "internship and residency" OR "internship" OR "house staff" OR "internship, medical" "medical internship" OR "medical internships" OR "medical residencies" OR "residencies" OR "residencies, medical" OR "residency" OR "residency, medical" OR "residency and internship" OR "staff, house" OR "surgical registrar" ) AND TITLE-ABS-KEY ( "orthopedic surgeon" OR "orthopedic surgeons" OR "orthopedist" OR "orthopedists" OR "surgeon, orthopedic" OR "surgeons, orthopedic" OR "orthopedics" OR "orthopaedic surgeon" OR "orthopaedic surgeons" OR "orthopaedist" OR "orthopaedists" OR "surgeon, orthopaedic" OR "surgeons, orthopaedic" OR "orthopaedics" ) | 973 |
