# Supplementary figures and images for: Barriers and facilitators for female practitioners in orthopaedic training and practice: a scoping review
Source: ANZ J Surg. 2025 Jan 3;95(4):647–57. doi: 10.1111/ans.19334 (PMC11982664; doi:10.1111/ans.19334)

**TABLE S3**: Mixed Methods Appraisal Tool24

**
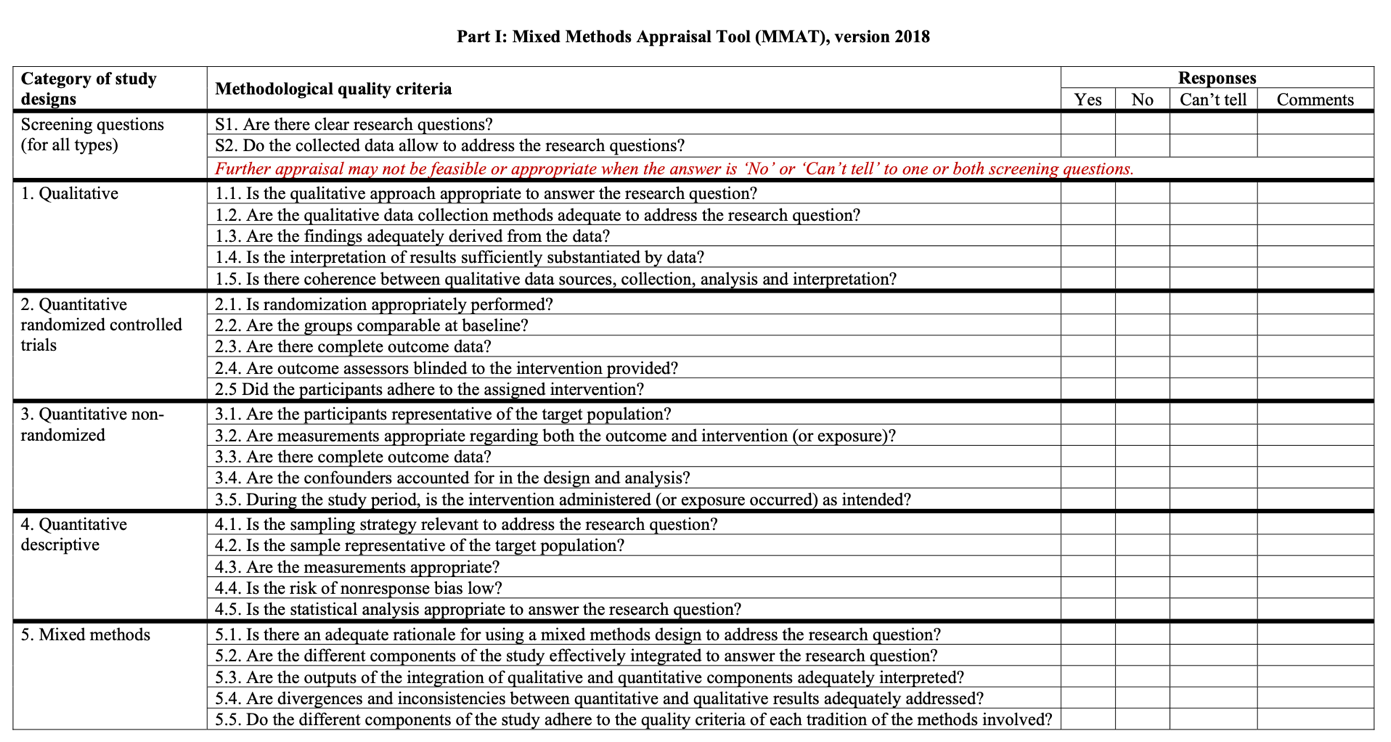
**

Supplement: Supplementary file 3 — Table S3. Mixed methods appraisal tool.24 [file ANS-95-647-s004.docx]
