# Supplementary material for: Barriers and facilitators for female practitioners in orthopaedic training and practice: a scoping review
Source: ANZ J Surg. 2025 Jan 3;95(4):647–57. doi: 10.1111/ans.19334 (PMC11982664; doi:10.1111/ans.19334)
Supplement: Supplementary file 4 — Table S4. Excluded studies during full text screening with reason. [file ANS-95-647-s003.docx]

**TABLE S4**: Excluded studies during full text screening with reason

**Excluded studies with reasons**

| **Author** | **Published Year** | **Reason for exclusion** |
| --- | --- | --- |
| Barnes | 2018 | Not specific to orthopaedic surgery |
| Berger-Groch | 2023 | Not specific to orthopaedic surgery |
| Buhren | 2021 | Not primary source |
| Chapman | 2019 | Not specific to orthopaedic surgery |
| Compton | 2021 | Not primary source |
| Farooq | 2009 | Population not relevant (medical students) |
| Galloway | 2022 | No data on gender discrepancies in orthopaedics |
| Guglielmetti | 2022 | No data on gender discrepancies in orthopaedics |
| Hoffmeister | 2019 | Not primary source |
| Hwang | 2021 | Not specific to orthopaedic surgery |
| Kim | 2020 | Abstract only article |
| Koech | 2023 | No data on gender discrepancies in orthopaedics |
| Lattanza | 2016 | Population not relevant (medical students) |
| Leong | 2024 | Subspecialty |
| Ortega | 2021 | No data on gender discrepancies in orthopaedics |
| Paulson | 2024 | Not primary source |
| Read | 2023 | Not specific to orthopaedic surgery |
| Shafiq | 2019 | No data on gender discrepancies in orthopaedics |
| Summers | 2020 | Not primary source |
| Wright | 2022 | Not primary source |
