# Supplementary material for: Barriers and facilitators for female practitioners in orthopaedic training and practice: a scoping review
Source: ANZ J Surg. 2025 Jan 3;95(4):647–57. doi: 10.1111/ans.19334 (PMC11982664; doi:10.1111/ans.19334)
Supplement: Supplementary file 5 — Table S5. characteristics of included studies. [file ANS-95-647-s005.docx]

**TABLE S5**: characteristics of included studies

| Study | | | | | | Participants | | | Interested outcome | |
| --- | --- | --- | --- | --- | --- | --- | --- | --- | --- | --- |
| Ref | First Author | Year | Country | Study design | Data collection source | n | Female identifying (%) | Stage of training | List of barriers identified | List of facilitators identified |
| 1^55^ | Alhammadi | 2024 | Saudi Arabia | Cross sectional survey | Orthopaedic surgeons | 96 | 100 | Consultants,  Trainees | 1. Microaggressions 2. Social exclusion 3. Negative attitudes and perceptions by surgical colleagues 4. Differential treatment from other hospital staff 5. Role misidentification 6. Gender discrimination e.g. missed opportunities due to gender 7. Gender stereotypes e.g. demeaning lecture material 8. Gender discrepancy in speaker roles at conferences 9. Disproportionate constraints (less leeway for mistakes, unequal scrutiny) 10. Devaluation, lack of deserved credit/ recognition 11. Tokenism 12. Gendered task assignment 13. Lacking facilities:  - locker rooms - pump/breastfeed - well-fitting lead shielding  1. Patient inflicted gender bias |  |
| 2^37^ | Alshammari | 2018 | Gulf Co-operation Council: Saudi Arabia, Kuwait, Oman | Cross sectional survey | Orthopaedic surgeons | 254 | 14.6 | Trainees | 1. Burnout 2. Marital status 3. Parental status 4. Physical demands 5. Negative attitudes and perceptions by surgical colleagues 6. Differential treatment by other healthcare workers 7. Gender discrimination 8. Gender inequality in career advancement/ promotion 9. Lower service volume 10. Gender based disparity in operative autonomy in training 11. Disproportionate constraints (less leeway for mistakes, unequal scrutiny, questioning) 12. Devaluation, lack of deserved credit/recognition | 1. Personal interest 2. Career satisfaction 3. Intellectual challenge |
| 3^81^ | Avila | 2023 | USA | Cross sectional analysis | Publicly available data | 19,006 | 5.6 | Consultants | 1. Financial reimbursement less for females 2. Lower service volume 3. Lower diversity of practice |  |
| 4^53^ | Balch Samora | 2020 | USA | Cross sectional survey | Orthopaedic surgeons | 264 | 100 | Consultants,  Trainee | 1. Microaggressions |  |
| 5^56^ | Balch Samora | 2020 | USA | Cross sectional survey | Orthopaedic surgeons | 927 | 67 | Consultants,  Trainees | 1. Bully 2. Sexual harassment 3. Gender discrimination 4. Lacking adequate structures and protocol to address discrimination, bullying and sexual harassment 5. Ineffective reporting streams |  |
| 6^69^ | Bauer | 2016 | USA | Cross sectional survey | Orthopaedic residency program directors | 1763 | 13.7 | Trainees | 1. Higher attrition rates |  |
| 7^82^ | Beebe | 2019 | USA | Retrospective cohort study | Orthopaedic surgeons | 10,114 | 5.2 | Consultants | 1. Financial reimbursement less for females 2. Lower service volume 3. Lower diversity of practice |  |
| 8^86^ | Bohl | 2019 | USA | Cross sectional survey | Orthopaedic surgeons | 488 | 100 | Consultants,  Trainees | 1. Illegal/ inappropriate interview questions 2. Ineffective reporting streams |  |
| 9^31^ | Brady | 2021 | USA | Retrospective cohort study | Orthopaedic surgeons | 41 | 28.6 | Trainees | 1. Competency bias, lower self-assessment |  |
| 10^73^ | Brook | 2020 | USA | Cross sectional survey | Orthopaedic surgeons | 117 | 18.8 | Consultants,  Trainees | 1. Gender based inequality to career advancement | 1. Mentorship |
| 11^59^ | Bucknall | 2009 | UK | Cross sectional survey | Male orthopaedic surgeons, patients, medical students | 545 | Not specified | n/a | 1. Negative attitudes and perceptions by male colleagues | 1. Patient preference and perceptions |
| 12^80^ | Buerba | 2020 | USA | Retrospective cohort study | Publicly available data | 22354 | 5.8 | Consultants | 1. Disproportionate research funding allocation 2. Financial reimbursement less |  |
| 13^38^ | Cafruni | 2022 | Argentina | Cross sectional mixed methods analysis | Administrative database, orthopaedic surgeons | 259 | 17 | Trainees | 1. Marital status 2. Parental status 3. Physical demands 4. Male dominated culture | 1. Personal interest 2. Career satisfaction 3. Enjoyment of manual labour 4. Positive medical school experiences |
| 14^97^ | DelPrete | 2023 | USA | Cross sectional survey | Orthopaedic surgeons | 207 | 100 | Consultants,  Trainees,  Medical students |  | 1. Mentorship 2. Networking 3. Social media |
| 15^83^ | Dossa | 2019 | Canada | Cross sectional analysis | Administrative database | 567 † | 7 † | Consultants,  Trainees | 1. Financial reimbursement less |  |
| 16^42^ | Downie | 2023 | UK | Retrospective case-control study | Administrative database | 274 | 33 | Trainees | 1. Longer parental leave required 2. Gender based disparity in operative autonomy 3. Increased rates of taking time off the training program for teaching, research, maternity |  |
| 17^33^ | Dupley | 2020 | UK | Cross sectional survey | Orthopaedic surgeons | 109 | 29 | Consultants,  Trainees | 1. Performance anxiety |  |
| 18^84^ | Forrester | 2020 | USA | Retrospective cohort study | Publicly available data | 2939 | 11 | Consultants | 1. Financial reimbursement less |  |
| 19^74^ | Gerull | 2020 | USA | Retrospective cohort study | Publicly available data | 3928 | 14 | Consultants | 1. Gender discrepancy in speaker roles at conferences 2. Gender imbalance in leadership roles and on committees | 1. Women in leadership positions and on committees 2. Stated diversity and inclusion efforts |
| 20^79^ | Gerull | 2021 | USA | Retrospective cohort study | Publicly available data | 794 | 8 | Consultants | 1. Underrepresentation in leadership awards 2. Implicit bias in unblinded awards process 3. Limited visibility | 1. Blinded award process |
| 21^70^ | Gerull | 2024 | USA | Retrospective cohort study | Administrative database | 4319 | 9.7 | Trainees | 1. Higher attrition rates |  |
| 22^57^ | Giglio | 2022 | Canada | Cross sectional survey | Orthopaedic surgeons | 465 | 28 | Consultants,  Trainees | 1. Sexual harassment 2. Gender based harassment |  |
| 23^88^ | Girgis | 2023 | USA | Retrospective case-control study | Orthopaedic residency program | 2113 | 16.77 | Applicants | 1. Letters of recommendation affinity bias |  |
| 24^65^ | Goss | 2020 | USA | Cross sectional survey | Orthopaedic surgeons | 158 | 100 | Trainees | 1. Lack of gender diverse faculty | 1. Trainee comradery 2. Clinical opportunities in training 3. Staff happiness |
| 25^98^ | Harbold | 2021 | USA | Retrospective cohort study | Junior doctors | 954 | 100 | Trainees |  | 1. Mentorship 2. Early exposure 3. Pipeline programs 4. Positive medical school experiences |
| 26^71^ | Haruno | 2023 | USA | Retrospective cohort study | Administrative database | 15,215 | 12.9 | Trainees | 1. Higher attrition rates | 1. Enhanced gender diversity |
| 27^28^ | Hiemstra | 2022 | Canada | Cross sectional mixed methods analysis | Administrative database, orthopaedic surgeons | 220 | 100 | Consultants,  Trainees,  Retired | 1. Imposter syndrome 2. Physical difficulty of pregnancy 3. Lack of role models and mentors 4. Bullying 5. Sexual harassment 6. Social exclusion 7. Negative attitudes and perceptions by surgical colleagues 8. Differential treatment from other hospital staff 9. Male dominated culture 10. Gender based inequality in career advancement 11. Gender imbalance in leadership roles and committees 12. Disproportionate constraints 13. Devaluation, lack deserved credit/ recognition 14. Gendered task assignments 15. Limited support networks 16. Societal norms: unequal child rearing responsibilities 17. Patient inflicted gender bias 18. Lack of or inadequate maternity leave policies and/or policy awareness |  |
| 28^35^ | Hiemstra | 2023 | Canada | Cross sectional survey | Orthopaedic surgeons | 218 | 100 | Consultants,  Trainees,  Retired | 1. Burnout 2. Male dominated culture 3. Disproportionate constraints 4. Devaluation | 1. Career satisfaction |
| 29^39^ | Higgins | 2021 | USA | Cross sectional survey | Orthopaedic surgeons | 377 | 23.9 | Consultants,  Trainees | 1. Marital status 2. Parental status 3. Gender based inequality to career advancement/promotion 4. Societal norms:  - House-hold workload - Childrearing responsibilities |  |
| 30^32^ | Hill | 2013 | USA | Cross sectional mixed methods analysis | Orthopaedic surgeons | 164 | 100 | Consultants | 1. Competency bias, lower self-assessment 2. Unsupportive partner 3. Lack of mentors 4. Gender stereotypes 5. Societal norms:  - House-hold workload - Childrearing responsibilities | 1. Career satisfaction 2. Desire to teach, mentor 3. Intellectual challenge 4. Mentorship |
| 31^48^ | Hill | 2013 | USA | Cross sectional mixed methods analysis | Administrative database, orthopaedic surgeons | 529 | 22 | Trainees | 1. Physical demands 2. Lack of mentors 3. Negative attitudes and perceptions by surgical colleagues | 1. Mentorship 2. Early exposure 3. Positive medical school experiences |
| 32^76^ | Hoof | 2020 | USA | Cross sectional analysis | Publicly available data | 4323 | 10 | Consultants | 1. Gender imbalance in leadership roles and committees | 1. Enhanced gender diversity |
| 33^60^ | Huntington | 2014 | USA | Cross sectional survey | Orthopaedic applicants | 207 | 15.5 | Applicants | 1. Negative attitudes and perceptions by surgical colleagues 2. Lack of gender diverse faculty 3. Illegal/inappropriate interview questions | 1. Trainee comradery 2. Positive medical school experience 3. Enhanced gender diversity 4. Positive interactions with staff 5. Staff happiness 6. Proximity to social support (support groups, family, friends) |
| 34^91^ | Incoll | 2021 | Australia | Retrospective cohort study | Administrative database | Not specified | Not specified | Applicants | 1. Lower training applicant scores | 1. Women on training selection panels 2. Stated diversity and inclusion efforts/ policy |
| 35^85^ | Jena | 2016 | USA | Cross sectional analysis | Publicly available data, administrative database | 233 † | 14.6 † | Consultants | 1. Financial reimbursement less |  |
| 36^101^ | Julian | 2023 | USA | Retrospective cohort study | Administrative database | 3624 | 19.2 | Trainees |  | 1. Women in leadership positions and on committees 2. Enhanced gender diversity (faculty + residents) 3. Dedicated women’s sports medicine programs |
| 37^49^ | Jurenovich | 2020 | USA | Cross sectional survey | Orthopaedic surgeons | 252 | 100 | Consultants,  Trainees | 1. Physical demands 2. Lack of female role models and mentors 3. Illegal/inappropriate interview questions | 1. Personal interest 2. Personal background as a sportsperson 3. Family member in surgery 4. Academic practice/ research |
| 38^89^ | Kobayashi | 2020 | USA | Cross sectional analysis | Orthopaedic residency program | 738 | 21 | Applicants | 1. Letters of recommendation affinity bias |  |
| 39^100^ | Kroin | 2019 | USA | Cross sectional survey | Orthopaedic applicants | 218 | 22.9 | Applicants |  | 1. Trainee comradery 2. Positive medical school experiences 3. Enhanced gender diversity 4. Positive interactions with staff 5. Staff happiness |
| 40^103^ | Levy | 2023 | USA | Cross sectional analysis | Orthopaedic residency programs | 172 ‡ | n/a | n/a |  | 1. Enhanced gender diversity |
| 41^64^ | London | 2016 | USA | Before and after, mixed methods study | Junior doctors | 58 | 31 | Trainees | 1. Lack of exposure to orthopaedics | 1. Early exposure 2. Positive medical school experiences |
| 42^50^ | Lurie | 2024 | USA | Cross sectional survey | Orthopaedic surgeons | 145 | 18.6 | Trainees | 1. Physical demands 2. Ineffective reporting streams 3. Ill-designed surgical instruments causing ergonomic challenges for smaller hands, less grip strength |  |
| 43^99^ | Mason | 2016 | USA | Retrospective cohort study | Junior doctors | 118 | 41 | Applicants |  | 1. Mentorship 2. Early exposure 3. Pipeline programs 4. Positive interactions with staff |
| 44^62^ | Meert | 2023 | Belgium | Cross sectional survey | Orthopaedic surgeons | 90 | 30 | Consultants,  Trainees | 1. Gender stereotypes | 1. Career satisfaction |
| 45^43^ | Mulcahey | 2019 | USA | Cross sectional survey | Orthopaedic surgeons | 190 | 100 | Trainees | 1. Deferring pregnancy 2. Pregnancy impact on scholarly activities 3. Longer parental leave required 4. Negative perceptions by surgical colleagues 5. Lacking facilities  - pump/ breast feeding - breast milk storage - childcare  1. Inadequate maternity leave and/or policy or policy awareness | 1. On site childcare 2. Facilities to breastfeed, pump and store milk 3. Formal standardised maternity/parental leave policies 4. Flexible scheduling |
| 46^104^ | Munger | 2018 | USA | Cross sectional analysis | Administrative database | 1. ‡ | 14.9 of applicants  13.2 of consultants | Consultants,  Applicants |  | 1. Enhanced gender diversity |
| 47^47^ | Nemeth | 2020 | USA | Cross sectional survey | Orthopaedic residency program directors | 61 | Not specified | Consultants,  Trainees | 1. Pregnancy impact on scholarly activities 2. Negative attitudes and perceptions by surgical colleagues 3. Pregnancy burden on co-trainees 4. Lacking facilities  - pump/ breast feeding - childcare  1. Inadequate maternity leave policy | 1. On site childcare 2. Facilities to breastfeed, pump and store milk 3. Formal standardised maternity/parental leave policies 4. Flexible scheduling |
| 48^95^ | Nguyen | 2020 | USA | Cross sectional survey | Orthopaedic surgeons | 801 | 100 | Consultants,  Trainees | 1. Financial burden of maternity leave especially for female consultants 2. Increase time taken off for research, teaching, maternity 3. Inadequate maternity leave policies | 1. On site childcare 2. Formal standardised maternity/ parental leave policies 3. Flexible scheduling |
| 49^75^ | Nwosu | 2023 | USA | Retrospective cohort study | Publicly available data | 3980 | 6.8 | Consultants | 1. Gender discrepancy in speaker roles at conferences 2. Gender imbalance in society leaderships and committees 3. Lack of visibility | 1. Enhanced gender diversity |
| 50^66^ | Okike | 2019 | USA | Cross sectional analysis | Administrative database | 450 | 100 | Consultants,  Trainees,  Applicants | 1. Lack of gender diverse faculty | 1. Enhanced gender diversity |
| 51^94^ | Peck | 2020 | USA | Cross sectional analysis | Publicly available data | 119 ‡ | n/a | Consultants | 1. Use of gendered terms reinforcing gender biases | 1. Gender neutral language |
| 52^63^ | Ponce | 2024 | USA | Cross sectional survey | Orthopaedic surgeons | 1125 | 12.7 | Consultants | 1. Workplace violence, physical threats and assaults |  |
| 53^40^ | Ponzio | 2022 | USA | Cross sectional survey | Orthopaedic surgeons | 347 | 44.1 | Consultants | 1. Marital status 2. Parental status 3. Gender imbalance in leadership roles and committees 4. Financial reimbursement less 5. Societal norms  - Household work - Childrearing responsibilities  1. Increase time off for maternity leave | 1. Career satisfaction |
| 54^92^ | Poon | 2019 | USA | Retrospective cohort study | Administrative database | 9133 applicants, 6381 enrolled residents | 12.6 - 16.0  (Applicant rate 2005-2016)  12.8 - 16.1*  (Trainee rate 2005-2016) | Trainees  Applicants | 1. Lower training application scores |  |
| 55^90^ | Powers | 2020 | USA | Cross sectional analysis | Administrative database | 730 | 21 | Applicants | 1. Letters of recommendation affinity bias 2. Lower training application scores | 1. Standardised letters of recommendation |
| 56^77^ | Ramos | 2022 | USA | Cross sectional survey | Orthopaedic society directors | 49 | n/a | Consultants | 1. Gender imbalance in leadership roles and committees | 1. Women in leadership positions and on committees |
| 57^45^ | Reid | 2021 | USA | Cross sectional survey | Orthopaedic surgeons, orthopaedic program directors | 495 | 23.8 | Trainees | 1. Deferring pregnancy 2. Pregnancy impact on scholarly activities 3. Negative attitudes and perceptions by surgical colleagues 4. Pregnancy burden on co-residents 5. Financial burden of maternity leave 6. Increased time taken off for maternity leave 7. Lack of maternity leave policies and/or policy awareness | 1. Formal maternity/ parental leave policies 2. On site childcare |
| 58^36^ | Rodarte | 2023 | USA | Cross sectional survey | Orthopaedic surgeons | 373 | 100 | Consultants,  Trainees | 1. Burnout 2. Gender discrimination - workplace conflict 3. Gender stereotypes  - Too bossy, assertive, demanding, difficult  1. Higher attrition rates 2. Gender based inequality to career advancement/ promotion 3. Disproportionate constraints (less leeway for mistakes, unequal scrutiny, written up) 4. Devaluation, lack of deserved credit/recognition | 1. Career satisfaction |
| 59^41^ | Rohde | 2016 | USA | Cross sectional survey | Orthopaedic surgeons | 232 | 100 | Consultants,  Trainees,  Retired | 1. Marital status 2. Parental status 3. Physical demands 4. Lack of role models and mentors 5. Lack of gender diverse faculty | 1. Career satisfaction 2. Intellectual challenge 3. Enjoyment of manual tasks 4. Mentorship 5. Early exposure |
| 60^46^ | Ruse | 2022 | USA | Cross sectional survey | Orthopaedic surgeons | 328 | 100 | Consultants,  Trainees,  Retired | 1. Deferred pregnancy 2. Negative attitudes and perceptions by surgical colleagues 3. Higher attrition rates 4. Limited support networks 5. Lacking facilities  - Pump/breast feeding - Milk storage - Childcare  1. Societal norms  - Child rearing responsibilities  1. Lack of inadequate maternity leave policies | 1. Onsite childcare 2. Facilities to breastfeed, pump and store milk 3. Formal, standardised maternity leave policies |
| 61^67^ | Sabesan | 2024 | USA | Retrospective cohort study | Administrative database | 3694 | 16.6 | Trainees | 1. Lack of gender diverse faculty 2. Lacking facilities  - childcare |  |
| 62^34^ | Sargent | 2011 | USA | Cross sectional survey | Orthopaedic surgeons | 648 | 9.9 | Consultants  Trainees | 1. Psychological distress 2. Burnout 3. Unsupportive partner 4. Lower marital harmony 5. Sexual harassment 6. Gender discrimination 7. Devaluation, lack of deserved credit/recognition |  |
| 63^102^ | Saxena | 2020 | USA | Cross sectional survey | Orthopaedic surgeons | 23 | n/a | Consultants |  | 1. Women in leadership positions and on committees 2. Enhanced gender diversity |
| 64^54^ | Sobel | 2023 | USA | Cross sectional survey | Orthopaedic surgeons | 94 | 16 | Trainees | 1. Microaggressions 2. Sexual harassment 3. Differential treatment from other hospital staff 4. Women were interrupted more when speaking 5. Illegal/inappropriate interview questions 6. Disproportionate constraints (unequal scrutiny) 7. Gendered task assignment 8. Patient inflicted gender bias |  |
| 65^78^ | Sobel | 2018 | USA | Cross sectional survey | Orthopaedics surgeons | 3,406 | 14.2 | Consultants  Trainees | 1. Gender imbalance in leadership positions | 1. Women in leadership positions 2. Academic practice/ research 3. Enhanced gender diversity 4. Dedicated women’s sports medicine program 5. Formalised, standardised maternity/paternity leave policies |
| 66^105^ | Stevens | 2023 | USA | Retrospective cohort study | Publicly available data | n/a | n/a | Consultants  Trainees |  | 1. Social media |
| 67^51^ | Tan | 2021 | New Zealand | Cross sectional mixed methods analysis | Orthopaedic surgeons, general surgeons | 821 | 65 | Consultants  Trainees  Juniors doctors  Medical students | 1. Physical demands 2. Lack of female role models 3. Gender discrimination 4. Negative attitudes and perceptions by surgical colleagues 5. Male dominated culture 6. Lack of exposure to orthopaedics 7. Societal norms  - Childrearing responsibilities | 1. Mentorship 2. Early exposure |
| 68^87^ | Theiss | 2022 | USA | Cross sectional survey | Orthopaedic applicants | 386 | 22.5 | Applicants | 1. Illegal/inappropriate interview questions |  |
| 69^29^ | Thiart | 2023 | South Africa | Qualitative descriptive study | Orthopaedic surgeons | 16 | 100 | Consultants  Trainees | 1. Financial reimbursement less 2. Lack of adequate maternity leave policies 3. Tokenism 4. Pride fatigue in challenging the status quo 5. Devaluation, lack of deserved credit/recognition 6. Deferring pregnancy 7. Pregnancy impact on scholarly activities 8. Pregnancy burden on co-residents 9. Societal norms:  - childrearing responsibilities  1. Social exclusion 2. Imposter syndrome/ non-belonging 3. Patient inflicted gender bias 4. Role misidentification 5. Differential treatment from other hospital staff 6. Women interrupted more when speaking 7. Illegal/inappropriate interview questions 8. Gender stereotypes e.g. crazy, emotional, sensitive, combative 9. Negative attitudes and perceptions from other hospital staff | 1. Stated diversity and inclusion efforts/ policy 2. Quota systems 3. Mentorship 4. Agency and pushing back against inequality 5. Career satisfaction |
| 70^30^ | Tosi | 1998 | USA | Cross sectional mixed methods analysis | Orthopaedic surgeons | 95 | 100 | Consultants  Trainees | 1. Imposter syndrome/ non-belonging 2. Marital status 3. Lack of mentors 4. Sexual harassment 5. Social exclusion 6. Negative attitudes and perceptions for surgical colleagues 7. Higher attrition rates 8. Gender based inequality to career advancement/ promotion 9. Gender discrepancy in speaker roles at conferences 10. Gender imbalance imbalances in leadership roles and committees 11. Limited support networks 12. Societal norms:  - household work - child-rearing responsibilities | 1. Desire to teach, mentor 2. Mentorship 3. Inclusion at social and sporting events 4. Trainee comradery 5. Pipeline programs 6. Women in leadership positions and on committees 7. Academic practice/ research 8. On site childcare 9. Flexible scheduling |
| 71^68^ | Vivekanatha | 2023 | Canada | Cross sectional analysis | Publicly available data | 1188 | 12.6 | Consultants | 1. Lack of gender diverse faculty 2. Gender discrepancy in speaker roles at conferences 3. Gender imbalance in leadership roles and committees 4. Limited visibility | 1. Women in leadership positions and on committees 2. Stated diversity and inclusion efforts/ policy |
| 72^72^ | Walker | 1993 | USA | Retrospective cohort study | Orthopaedic residency directors | 2225 | 4.7 | Trainees | 1. Higher attrition rate for women |  |
| 73^106^ | Wang | 2022 | USA | Cross sectional analysis | Publicly available data | n/a | n/a | Trainees |  | 1. Social media |
| 74^93^ | Webber | 2022 | USA | Cross sectional analysis | Administrative data | 320 | 15.3 | Applicants | 1. Lower training application scores | 1. Higher training interview scores |
| 75^58^ | Whicker | 2020 | USA | Cross sectional survey | Orthopaedic surgeons | 250 | 100 | Consultants  Trainees | 1. Sexual harassment 2. Lacking inadequate structures and protocol to address sexual harassment 3. Ineffective reporting streams |  |
| 76^96^ | Wynn | 2021 | USA | Cross sectional analysis | Orthopaedic residency directors | n/a | n/a | Trainees | 1. Lack of facilities  - Pump/ breastfeeding - Milk storage  1. Lack of breastfeeding policies | 1. Enhanced gender diversity |
| 77^44^ | Wynn | 2022 | USA | Qualitative descriptive study | Orthopaedic surgeons | 4 | 100 | Consultants | 1. Physical difficulties of pregnancy 2. Longer parental leave required 3. Lacking facilities  - Childcare - Pump/breastfeeding  1. Societal norms:  - Childbearing responsibilities  1. Inadequate maternity leave policies and/or policy awareness | 1. Agency e.g. adequate hydration and OR temperature control during pregnancy 2. Supportive partner 3. Supportive colleagues 4. Formal standardised maternity/paternity leave policy 5. Flexible scheduling |
| 78^52^ | Xu | 2023 | USA and inter-national | Cross sectional survey | Orthopaedic surgeons | 282 | 100 | Consultants  Trainees  Medical students | 1. Lack of female role models and mentors 2. Sexual harassment 3. Male dominated culture 4. Lack of gender diverse faculty 5. Gender based inequality to career advancement/ promotion 6. Gender imbalance in leadership roles and committees 7. Societal norms  - Household work - Childrearing responsibilities  1. Patient inflicted gender bias |  |
| 79^61^ | Yonai | 2023 | Israel | Cross sectional survey | Orthopaedic surgeons | 149 | 100 | Trainees | 1. Gender discrimination | 1. Personal interest 2. Career satisfaction 3. Early exposure 4. Positive medical school experiences |
| † Study contained data for various surgical specialties. Only the orthopaedic surgery participants and their data is displayed and used  ‡ Data on departments/programs, not participants | | | | | | | | | | |
