# Supplementary material for: Barriers and facilitators for female practitioners in orthopaedic training and practice: a scoping review
Source: ANZ J Surg. 2025 Jan 3;95(4):647–57. doi: 10.1111/ans.19334 (PMC11982664; doi:10.1111/ans.19334)
Supplement: Supplementary file 6 — Table S6. Barriers and facilitators for female practitioners in orthopaedic surgery mapped to the socio‐ecological model. [file ANS-95-647-s007.docx]

**TABLE S6:** Barriers and facilitators for female practitioners in orthopaedic surgery mapped to the Socio-Ecological Model

| **LEVEL** | **BARRIERS** | **FACILITATORS** |
| --- | --- | --- |
| **Individual** | **Psychological**   - Imposter syndrome/non-belonging^28-30^ - Competency bias, lower self-assessment^31, 32^ - Performance anxiety^32, 33^ - Psychological distress^34^ - Burnout^34-37^ - Pride fatigue in challenging the status quo^29^   **Personal life**   - Unsupportive partner^32, 34^ - Lower marital harmony^34^ - Marital status^30, 37-41^ - Parental status^37-41^ - Longer parental leave required^42-44^   **Physical**   - Physical demands^37, 38, 41, 48-51^ - Deferring pregnancy^29, 43, 45, 46^ - Pregnancy impact on scholarly activities^29, 43, 45, 47^ - Physical difficulty of pregnancy^28, 44^ | **Psychological**   - Personal interest^37, 38, 49, 61^ - Career satisfaction^29, 32, 35-38, 40, 41, 61, 62^ - Desire to teach, mentor^30, 32^ - Intellectual challenge^32, 37, 41^   **Personal life**   - Personal background as a sportsperson^49^ - Family member practicing surgery^49^ - Agency and pushing back against inequality^29, 44^ - Supportive partner^44^   **Physical**   - Enjoyment of manual tasks^38, 41^ |
| **Interpersonal** | **Support systems**   - Lack of role models and mentors^28, 30, 32, 41, 48, 49, 51, 52^   **Workplace culture**   - Microaggressions^53-55^ - Bullying^28, 56^ - Sexual harassment^28, 30, 34, 52, 54, 56-58^ - Gender-based harassment^57^ - Social exclusion^28-30, 55^   **Professional relationships**   - Negative attitudes and perceptions by surgical colleagues^28-30, 37, 43, 45-48, 51, 55, 59, 60^ - Differential treatment from other hospital staff^28, 29, 37, 54, 55^ - Role misidentification^29, 55^ - Women were interrupted more when speaking^29, 54^ - Pregnancy burden on co-trainees^29, 43, 45, 47^ | **Support systems**   - Mentorship^29, 30, 32, 41, 48, 51, 73, 97-99^ - Networking^97^ - Supportive colleagues^44^   **Workplace culture**   - Inclusion at sporting and social events^30^   **Professional relationships**   - Trainee comradery^30, 60, 65, 100^ |
| **Organisational** | **Discrimination and bias**   - Gender discrimination^34, 36, 37, 51, 55, 56, 61^ - Gender stereotypes including labelling; bossy, demanding, pushy, difficult etc^29, 32, 36, 55, 62^ - Workplace violence, physical threats and assaults^63^ - Male dominated culture^28, 35, 38, 51, 52^   **Career advancement**   - Lack of exposure to orthopaedics^51, 64^ - Lack of gender diverse faculty^41, 52, 60, 65-68^ - Higher attrition rates^30, 36, 46, 69-72^ - Gender based inequality to career advancement/ promotion^28, 30, 36, 37, 39, 52, 73^ - Gender discrepancy in speaker roles at conferences^30, 55, 68, 74, 75^ - Gender imbalance in leadership roles and committees^28, 30, 40, 52, 68, 74-78^ - Underrepresentation in leadership awards^79^ - Implicit bias in unblinded awards processes^79^ - Disproportionate research funding allocation^80^ - Financial reimbursement less^29, 40, 80-85^   **Work environment**   - Illegal/ inappropriate interview questions^29, 49, 54, 60, 86, 87^ - Letters of recommendation affinity bias^88-90^ - Lower training application scores^90-93^ - Gender based disparity in operative autonomy in training^37, 42^ - Lower service volume^37, 81, 82^ - Lower diversity of practice^81, 82^ - Disproportionate constraints (less leeway for mistakes, unequal scrutiny, questioning)^28, 35-37, 54, 55^ - Devaluation, lack of deserved credit/recognition^28, 29, 34-37, 55^ - Tokenism^29, 55^ - Gendered task assignment^28, 54, 55^ - Use of gender-specific terms reinforcing gender biases such as chairman^94^ - Limited support networks^28, 30, 46^   **Facilities and organisational strategies**   - Lacking facilities^43, 44, 46, 47, 55, 67, 96^ - Locker rooms^55^ - Well-fitting lead shielding^55^ - Pump/breast feeding^43, 44, 46, 47, 55, 96^ - Milk storage^43, 46, 96^ - Childcare ^43, 44, 46, 47, 67^ - Financial burden of maternity leave ^45, 95^ - Increased time taken off whether for teaching, research and/or maternity leave^40, 42, 45, 95^ | **Discrimination and bias**   - Gender neutral language such as chairperson^94^   **Career advancement**   - Early exposure^41, 48, 51, 61, 64, 98, 99^ - Pipeline programs^30, 98, 99^ - Positive medical school experiences^38, 48, 60, 61, 64, 98, 100^ - Clinical opportunities in training^65^ - Women in leadership positions and on committees^30, 68, 74, 77, 78, 101, 102^ - Higher training interview scores^91, 93^ - Academic practice/ research^30, 49, 78^   **Work environment**   - Enhanced gender diversity^60, 66, 71, 75, 76, 78, 96, 100-104^ - Positive interactions with staff^60, 99, 100^ - Staff happiness^60, 65, 100^ - On site childcare^30, 43, 45-47, 95^ - Facilitates to breastfeed, pump and store milk^43, 46, 47^   **Facilities and organisational strategy**   - Quota systems^29^ - Standardised letters of recommendation^90^ - Women on training selection panels^91^ - Dedicated women’s sports medicine programs^78, 101^ - Blinded award process^79^ |
| **Community** | - Limited visibility^68, 75, 79^ - Societal norms and attitudes:^28, 39^ ^29, 30, 32, 40, 46, 51, 52^ - Household workload^30, 32, 39, 40, 52^ - Child -rearing responsibilities^28-30, 32, 39, 40, 44, 46, 51, 52^ - Patient inflicted gender bias^28, 29, 52, 54, 55^ | - Social media^97, 105, 106^ - Proximity to social support - support groups, family, friends^60^ - Patient preference and perceptions^59^ |
| **Policy** | - Lacking adequate structures and protocol to address discrimination, bullying and sexual harassment^56, 58^ - Lack of or inadequate maternity leave policies and/or policy awareness^28, 29, 43-47, 95^ - Lack of breastfeeding policies^96^ - Ineffective reporting streams^50, 56, 58, 86^ - Ill-designed surgical instruments causing ergonomic challenges for smaller hands, less grip strength^50^ | - Stated diversity and inclusion efforts/policy^29, 68, 74, 91^ - Formal, standardised maternity/parental leave policies^43-47, 78, 95^ - Flexible scheduling^30, 43, 44, 47, 95^ |
| **No category** | - Women typically employed at hospitals more than in private practice^40, 41^ | |
