# Supplementary material for: Barriers and facilitators for female practitioners in orthopaedic training and practice: a scoping review
Source: ANZ J Surg. 2025 Jan 3;95(4):647–57. doi: 10.1111/ans.19334 (PMC11982664; doi:10.1111/ans.19334)
Supplement: Supplementary file 7 — Table S7. Quality assessment of included studies using the MMAT.24 [file ANS-95-647-s001.docx]

**TABLE S7**: Quality assessment of included studies using the MMAT^24^

| Design (number) | Quality criteria | Results |
| --- | --- | --- |
| Screening (all types, n=79) | Clear research question; data collected addresses research question | Studies adequately addressed screening questions |
| Qualitative (2) | Qualitative approach appropriateness; collection methods adequate; findings adequately derived from data; sufficient substantiation of interpretation by data; coherence between qualitative data sources, collection, analysis and interpretation | Studies adequately addressed criteria |
| Quantitative randomised control trial (0) | Appropriately performed randomisation; groups comparable at baseline; complete outcome data; outcome assessors blinded to intervention; participants adhered to assigned intervention | n/a |
| Quantitative non-randomised control trial (32) | Participants representative of the target population; appropriateness of measurements; completeness of outcome data; confounders accounted for; exposure occurred as intended | 9 studies included other populations aside from orthopaedic surgeons. These included orthopaedic trainee position applicants and orthopaedic program directors reporting on behalf of their programs. 3 studies did not account for confounding factors. 1 studied lacked complete data outcome. Notably this study also was not completed by the target population and did not account for confounding. All other studies adequately addressed the other criteria. Suboptimal quality criteria was noted for 11 studies. |
| Quantitative descriptive (38) | Sampling strategy relevant; sample representative of the target population; measurements appropriate; risk of nonresponse bias; statistical analysis appropriate | 8 studies were at risk of nonresponse bias and 9 scored a cannot tell as they did not state the response rate. 5 studies included other populations aside from orthopaedic surgeons. These included orthopaedic trainee position applicants and orthopaedic program directors reporting on behalf of their programs. All the studies adequately addressed other criteria. Suboptimal adherence to the quality criteria was noted in 20 studies. |
| Mixed methods (7) | Rationale adequate; effective integration of components; adequate addressal of divergences and inconsistencies between components; adherence to quality criteria of component study designs | All studies par one adequately addressed the criteria. The study which did not had a larger female participant proportion then that of the target population |
