# Supplementary material for: Barriers and facilitators for female practitioners in orthopaedic training and practice: a scoping review
Source: ANZ J Surg. 2025 Jan 3;95(4):647–57. doi: 10.1111/ans.19334 (PMC11982664; doi:10.1111/ans.19334)
Supplement: Supplementary file 8 — Table S8. Quality assessment of included studies using the mixed methods appraisal tool.24 [file ANS-95-647-s006.docx]

**TABLE S8:** Quality assessment of included studies using the Mixed Methods Appraisal tool^24^

|  | 1. Qualitative | | | | | 2. Quantitative RCT | | | | | 3. Quantitative non-RCT | | | | | 4. Quantitative descriptive | | | | | 5. Mixed methods | | | | |
| --- | --- | --- | --- | --- | --- | --- | --- | --- | --- | --- | --- | --- | --- | --- | --- | --- | --- | --- | --- | --- | --- | --- | --- | --- | --- |
| Author | 1.1 | 1.2 | 1.3 | 1.4 | 1.5 | 2.1 | 2.2 | 2.3 | 2.4 | 2.5 | 3.1 | 3.2 | 3.3 | 3.4 | 3.5 | 4.1 | 4.2 | 4.3 | 4.4 | 4.5 | 5.1 | 5.2 | 5.3 | 5.4 | 5.5 |
| Alhammadi |  |  |  |  |  |  |  |  |  |  |  |  |  |  |  | Y | Y | Y | C | Y |  |  |  |  |  |
| Alshammari |  |  |  |  |  |  |  |  |  |  |  |  |  |  |  | Y | Y | Y | Y | Y |  |  |  |  |  |
| Avila |  |  |  |  |  |  |  |  |  |  | Y | Y | Y | Y | Y |  |  |  |  |  |  |  |  |  |  |
| Balch Samora |  |  |  |  |  |  |  |  |  |  |  |  |  |  |  | Y | Y | Y | Y | Y |  |  |  |  |  |
| Balch Samora |  |  |  |  |  |  |  |  |  |  |  |  |  |  |  | Y | Y | Y | N | Y |  |  |  |  |  |
| Bauer |  |  |  |  |  |  |  |  |  |  |  |  |  |  |  | Y | Y | Y | Y | Y |  |  |  |  |  |
| Beebe |  |  |  |  |  |  |  |  |  |  | Y | Y | Y | Y | Y |  |  |  |  |  |  |  |  |  |  |
| Bohl |  |  |  |  |  |  |  |  |  |  |  |  |  |  |  | Y | Y | Y | Y | Y |  |  |  |  |  |
| Brady |  |  |  |  |  |  |  |  |  |  | Y | Y | Y | Y | Y |  |  |  |  |  |  |  |  |  |  |
| Brook |  |  |  |  |  |  |  |  |  |  |  |  |  |  |  | Y | Y | Y | Y | Y |  |  |  |  |  |
| Bucknall |  |  |  |  |  |  |  |  |  |  |  |  |  |  |  | Y | N | Y | Y | Y |  |  |  |  |  |
| Buerba |  |  |  |  |  |  |  |  |  |  | Y | Y | Y | Y | Y |  |  |  |  |  |  |  |  |  |  |
| Cafruni |  |  |  |  |  |  |  |  |  |  |  |  |  |  |  |  |  |  |  |  | Y | Y | Y | Y | Y |
| DelPrete |  |  |  |  |  |  |  |  |  |  |  |  |  |  |  | Y | Y | Y | Y | Y |  |  |  |  |  |
| Downie |  |  |  |  |  |  |  |  |  |  | Y | Y | Y | Y | Y |  |  |  |  |  |  |  |  |  |  |
| Dossa |  |  |  |  |  |  |  |  |  |  | Y | Y | Y | Y | Y |  |  |  |  |  |  |  |  |  |  |
| Dupley |  |  |  |  |  |  |  |  |  |  |  |  |  |  |  | Y | Y | Y | Y | Y |  |  |  |  |  |
| Forrester |  |  |  |  |  |  |  |  |  |  | Y | Y | Y | Y | Y |  |  |  |  |  |  |  |  |  |  |
| Gerull |  |  |  |  |  |  |  |  |  |  | Y | Y | Y | Y | Y |  |  |  |  |  |  |  |  |  |  |
| Gerull |  |  |  |  |  |  |  |  |  |  | Y | Y | Y | Y | Y |  |  |  |  |  |  |  |  |  |  |
| Gerull |  |  |  |  |  |  |  |  |  |  | Y | Y | Y | Y | Y |  |  |  |  |  |  |  |  |  |  |
| Giglio |  |  |  |  |  |  |  |  |  |  |  |  |  |  |  | Y | Y | Y | N | Y |  |  |  |  |  |
| Girgis |  |  |  |  |  |  |  |  |  |  | N | Y | Y | Y | Y |  |  |  |  |  |  |  |  |  |  |
| Goss |  |  |  |  |  |  |  |  |  |  |  |  |  |  |  | Y | Y | Y | Y | Y |  |  |  |  |  |
| Harbold |  |  |  |  |  |  |  |  |  |  | N | Y | N | N | Y |  |  |  |  |  |  |  |  |  |  |
| Haruno |  |  |  |  |  |  |  |  |  |  | Y | Y | Y | Y | Y |  |  |  |  |  |  |  |  |  |  |
| Hiemstra |  |  |  |  |  |  |  |  |  |  |  |  |  |  |  |  |  |  |  |  | Y | Y | Y | Y | Y |
| Hiemstra |  |  |  |  |  |  |  |  |  |  |  |  |  |  |  | Y | Y | Y | Y | Y |  |  |  |  |  |
| Higgins |  |  |  |  |  |  |  |  |  |  |  |  |  |  |  | Y | Y | Y | N | Y |  |  |  |  |  |
| Hill |  |  |  |  |  |  |  |  |  |  |  |  |  |  |  |  |  |  |  |  | Y | Y | Y | Y | Y |
| Hill |  |  |  |  |  |  |  |  |  |  |  |  |  |  |  |  |  |  |  |  | Y | Y | Y | Y | Y |
| Hoof |  |  |  |  |  |  |  |  |  |  | Y | Y | Y | Y | Y |  |  |  |  |  |  |  |  |  |  |
| Huntington |  |  |  |  |  |  |  |  |  |  |  |  |  |  |  | Y | N | Y | Y | Y |  |  |  |  |  |
| Incoll |  |  |  |  |  |  |  |  |  |  | Y | Y | Y | Y | Y |  |  |  |  |  |  |  |  |  |  |
| Jena |  |  |  |  |  |  |  |  |  |  | Y | Y | Y | Y | Y |  |  |  |  |  |  |  |  |  |  |
| Julian |  |  |  |  |  |  |  |  |  |  | Y | Y | Y | Y | Y |  |  |  |  |  |  |  |  |  |  |
| Jurenovich |  |  |  |  |  |  |  |  |  |  |  |  |  |  |  | Y | Y | Y | C | Y |  |  |  |  |  |
| Kobayashi |  |  |  |  |  |  |  |  |  |  | N | Y | Y | Y | Y |  |  |  |  |  |  |  |  |  |  |
| Kroin |  |  |  |  |  |  |  |  |  |  |  |  |  |  |  | Y | N | Y | N | Y |  |  |  |  |  |
| Levy |  |  |  |  |  |  |  |  |  |  | N | Y | Y | Y | Y |  |  |  |  |  |  |  |  |  |  |
| London |  |  |  |  |  |  |  |  |  |  |  |  |  |  |  |  |  |  |  |  | Y | Y | Y | Y | Y |
| Lurie |  |  |  |  |  |  |  |  |  |  |  |  |  |  |  | Y | Y | Y | N | Y |  |  |  |  |  |
| Mason |  |  |  |  |  |  |  |  |  |  | N | Y | Y | Y | Y |  |  |  |  |  |  |  |  |  |  |
| Meert |  |  |  |  |  |  |  |  |  |  |  |  |  |  |  | Y | Y | Y | C | Y |  |  |  |  |  |
| Mulcahey |  |  |  |  |  |  |  |  |  |  |  |  |  |  |  | Y | Y | Y | Y | Y |  |  |  |  |  |
| Munger |  |  |  |  |  |  |  |  |  |  | N | Y | Y | Y | Y |  |  |  |  |  |  |  |  |  |  |
| Nemeth |  |  |  |  |  |  |  |  |  |  |  |  |  |  |  | Y | Y | Y | C | Y |  |  |  |  |  |
| Nguyen |  |  |  |  |  |  |  |  |  |  |  |  |  |  |  | Y | Y | Y | Y | Y |  |  |  |  |  |
| Nwosu |  |  |  |  |  |  |  |  |  |  | Y | Y | Y | Y | Y |  |  |  |  |  |  |  |  |  |  |
| Okike |  |  |  |  |  |  |  |  |  |  | Y | Y | Y | Y | Y |  |  |  |  |  |  |  |  |  |  |
| Peck |  |  |  |  |  |  |  |  |  |  | Y | Y | Y | Y | Y |  |  |  |  |  |  |  |  |  |  |
| Ponce |  |  |  |  |  |  |  |  |  |  |  |  |  |  |  | Y | Y | Y | N | Y |  |  |  |  |  |
| Ponzio |  |  |  |  |  |  |  |  |  |  |  |  |  |  |  | Y | Y | Y | C | Y |  |  |  |  |  |
| Poon |  |  |  |  |  |  |  |  |  |  | Y | Y | Y | Y | Y |  |  |  |  |  |  |  |  |  |  |
| Powers |  |  |  |  |  |  |  |  |  |  | Y | Y | Y | Y | Y |  |  |  |  |  |  |  |  |  |  |
| Ramos |  |  |  |  |  |  |  |  |  |  |  |  |  |  |  | Y | N | Y | Y | Y |  |  |  |  |  |
| Reid |  |  |  |  |  |  |  |  |  |  |  |  |  |  |  | Y | Y | Y | N | Y |  |  |  |  |  |
| Rodarte |  |  |  |  |  |  |  |  |  |  |  |  |  |  |  | Y | Y | Y | C | Y |  |  |  |  |  |
| Rhode |  |  |  |  |  |  |  |  |  |  |  |  |  |  |  | Y | Y | Y | Y | Y |  |  |  |  |  |
| Ruse |  |  |  |  |  |  |  |  |  |  |  |  |  |  |  | Y | Y | Y | C | Y |  |  |  |  |  |
| Sabesan |  |  |  |  |  |  |  |  |  |  | Y | Y | Y | N | Y |  |  |  |  |  |  |  |  |  |  |
| Sargent |  |  |  |  |  |  |  |  |  |  |  |  |  |  |  | Y | Y | Y | C | Y |  |  |  |  |  |
| Saxena |  |  |  |  |  |  |  |  |  |  |  |  |  |  |  | Y | Y | Y | Y | Y |  |  |  |  |  |
| Sobel |  |  |  |  |  |  |  |  |  |  |  |  |  |  |  | Y | Y | Y | Y | Y |  |  |  |  |  |
| Soebl |  |  |  |  |  |  |  |  |  |  |  |  |  |  |  | Y | Y | Y | Y | Y |  |  |  |  |  |
| Stevens |  |  |  |  |  |  |  |  |  |  | Y | Y | Y | N | Y |  |  |  |  |  |  |  |  |  |  |
| Tan |  |  |  |  |  |  |  |  |  |  |  |  |  |  |  |  |  |  |  |  | Y | Y | Y | Y | N |
| Theiss |  |  |  |  |  |  |  |  |  |  |  |  |  |  |  | Y | N | Y | N | Y |  |  |  |  |  |
| Thiart | Y | Y | Y | Y | Y |  |  |  |  |  |  |  |  |  |  |  |  |  |  |  |  |  |  |  |  |
| Tosi |  |  |  |  |  |  |  |  |  |  |  |  |  |  |  |  |  |  |  |  | Y | Y | Y | Y | Y |
| Vivekanatha |  |  |  |  |  |  |  |  |  |  | Y | Y | Y | Y | Y |  |  |  |  |  |  |  |  |  |  |
| Walker |  |  |  |  |  |  |  |  |  |  |  |  |  |  |  | Y | Y | Y | Y | Y |  |  |  |  |  |
| Wang |  |  |  |  |  |  |  |  |  |  | N | Y | Y | Y | Y |  |  |  |  |  |  |  |  |  |  |
| Webber |  |  |  |  |  |  |  |  |  |  | N | Y | Y | Y | Y |  |  |  |  |  |  |  |  |  |  |
| Whicker |  |  |  |  |  |  |  |  |  |  |  |  |  |  |  | Y | Y | Y | Y | Y |  |  |  |  |  |
| Wynn |  |  |  |  |  |  |  |  |  |  | N | Y | Y | Y | Y |  |  |  |  |  |  |  |  |  |  |
| Wynn | Y | Y | Y | Y | Y |  |  |  |  |  |  |  |  |  |  |  |  |  |  |  |  |  |  |  |  |
| Xu |  |  |  |  |  |  |  |  |  |  |  |  |  |  |  | Y | Y | Y | C | Y |  |  |  |  |  |
| Yonai |  |  |  |  |  |  |  |  |  |  |  |  |  |  |  | Y | Y | Y | Y | Y |  |  |  |  |  |
| Y: Yes  N: No  C: Can’t Tell | | | | | | | | | | | | | | | | | | | | | | | | | |
